# Dietary Interventions for Sleep Health: Multi‐Population and Mendelian Randomization Evidence on Sleep Outcomes and Disorders

**DOI:** 10.1002/fsn3.71475

**Published:** 2026-02-10

**Authors:** Meixiu Lin, Siliang Ge, Kaiweisa Abuduxukuer, Yingfan Chen, Shanshan Yang, Ke Han, Ming Chen

**Affiliations:** ^1^ Shanghai Key Laboratory of Craniomaxillofacial Development and Diseases Shanghai China; ^2^ Department of Orthodontics Shanghai Stomatological Hospital & School of Stomatology, Fudan University Shanghai China; ^3^ Department of Orthopedics The Fourth Medical Center of Chinese PLA General Hospital Beijing China; ^4^ National Clinical Research Center for Orthopedics, Sports Medicine & Rehabilitation Beijing China; ^5^ Department of Biostatistics, School of Public Health Fudan University Shanghai China; ^6^ Department of Traditional Chinese Medicine The Sixth Medical Center of Chinese PLA General Hospital Beijing China; ^7^ Graduate School of Chinese PLA General Hospital Beijing China; ^8^ Department of Endoscopy General Hospital of Northern Theater Command Shenyang China; ^9^ National Clinical Research Center for Geriatrics Diseases Beijing China; ^10^ Institute of Geriatrics, Beijing Key Laboratory of Aging and Geriatrics, Chinese PLA General Hospital Beijing China

## Abstract

Sleep disorders represent major public health concerns with significant health consequences. While diet shows promise as a modifiable intervention, the differential effects of established dietary patterns on specific sleep phenotypes and the contributions of specific food groups remains unclear. Therefore, this study investigated how three established dietary patterns relate to distinct sleep outcomes, and further explored the contributions of specific food components. We combined four decades of data and analyzed 9040 US adults from NHANES with sampling weights. Five sleep outcomes were examined in relation to dietary pattern adherence, quantified through averaged 24‐h recall data from two consecutive days: including self‐reported sleep duration, sufficiency, OSA, daytime sleepiness, and stop breathing. For international validation, we used the Global Dietary Database (62 countries) linked to country‐level sleep apnea prevalence (191 countries). Cohort analysis employed the Chinese Longitudinal Healthy Longevity Survey (*n* = 6885). Causal associations between dietary components and sleep phenotypes were investigated using two‐sample Mendelian randomization with genome‐wide association summary statistics. Our results demonstrated that greater the Mediterranean Diet (MED) adherence was associated with longer sleep duration (*β* = 0.06, 95% CI: 0.03–0.10, *p* < 0.001) and sufficient sleep (OR = 1.10, 95% CI: 1.04–1.15, *p* < 0.001). Notably, dietary Approaches to Stop Hypertension (DASH) adherence offered the strongest protection against daytime sleepiness (OR = 0.89, 95% CI: 0.85–0.93, *p* < 0.001) and breathing cessation episodes (OR = 0.91, 95% CI: 0.85–0.96, *p* = 0.001). The Alternative Healthy Eating Index (AHEI) demonstrated balanced associations with improved sleep sufficiency (OR = 1.006, *p* < 0.05), reduced daytime sleepiness (OR = 0.995, *p* < 0.05), and fewer breathing episodes (OR = 0.989, *p* < 0.01). Furthermore, food‐component‐level analyses revealed consistent protective associations from legumes, nuts, and whole grains, while vegetables, fruits, meats, and SSBs showed variable effects—findings supported by both multi‐database analyses and Mendelian randomization. Importantly, MR further demonstrated specific causal associations for fruits and vegetables: pears and strong vegetable preferences protected against short sleep, while cabbage, grapefruit, and melon causally increased sleep disorder risk. Additionally, processed meats causally increased OSA and snoring risk, with food processing degree emerging as a critical causal determinant. In conclusion, this study makes three main contributions. First, we provide a direct comparison of multiple dietary patterns (MED, DASH, AHE) across diverse sleep phenotypes. Second, we identify specific protective food components, with genetic causal evidence supporting their effects on sleep. Third, we integrate multi‐national datasets to validate findings across diverse populations, substantially enhancing generalizability. Collectively, these findings support evidence‐based dietary interventions for sleep health improvement.

## Graphical abstract

This multi‐population study examined three dietary patterns (MED, DASH, AHE) across distinct sleep phenotypes using NHANES data (*n* = 9040), global validation (62 countries), Chinese cohort replication (*n* = 6885), and Mendelian randomization for causal inference. Mediterranean Diet adherence significantly improved sleep duration and sufficiency, while DASH offered the strongest protection against sleep‐disordered breathing. Food‐component analyses revealed consistent benefits from legumes, nuts, and whole grains, with the degree of food processing critically determining sleep disorder risk. Findings support phenotype‐specific dietary interventions for precision sleep health management.

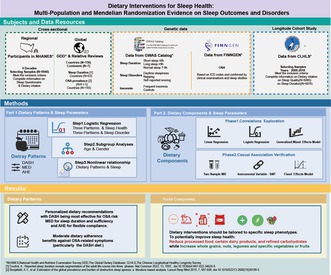

## 1 Introduction

Sleep disturbances have emerged as critical determinants of population health. Conditions such as obstructive sleep apnea (OSA), insomnia, daytime sleepiness, and insufficient nocturnal sleep affect multiple physiological systems and contribute to chronic disease risk (Potts et al. 2024; Robbins and Quan 2024; Xiao et al. 2025). Chronic sleep disturbances substantially impair quality of life and increase risks of cardiovascular disease, metabolic dysfunction, and psychiatric morbidity (Potts et al. 2024; Xiao et al. 2025). Given these serious health implications, researchers are investigating feasible lifestyle interventions to improve sleep quality, such as exercise habits, nutritional tweaks, and changes to one's surroundings. Among these, dietary patterns show promise as a modifiable, accessible, and adherable intervention (Potts et al. 2024; Xiao et al. 2025).

Accumulating evidence from observational and interventional studies suggests that high‐quality dietary patterns may significantly improve sleep outcomes (Arab et al. 2024; Kechribari et al. 2022; Papandreou et al. 2012; Potts et al. 2024; Xiao et al. 2025). Evidence‐based dietary patterns, such as the Mediterranean diet (MED), Dietary Approaches to Stop Hypertension (DASH), and Alternative Healthy Eating (AHE), have gained attention for their sleep benefits: MED adherence is associated with reduced insomnia risk and improved sleep quality scores (Papandreou et al. 2012; Potts et al. 2024; Xiao et al. 2025). Similarly, DASH and AHE compliance correlates with better sleep efficiency and reduced OSA severity (Chiuve et al. 2012; Li et al. 2023; Potts et al. 2024; Rokou et al. 2023; Xiao et al. 2025; Zhao et al. 2024). These patterns encourage high intake of minimally processed plant foods—whole grains, legumes, nuts, vegetables, fruits—and unsaturated fats, while discouraging consumption of red and processed meats, saturated fats, and added sugars. Researchers found that they can help sleep by reducing body inflammation and promoting metabolism (Chen et al. 2023; Potts et al. 2024, 2023; Xiao et al. 2025).

Despite these promising findings, the relationship between comprehensive dietary patterns, specific food components, and distinct sleep parameters remains insufficiently characterized. Current research exhibits several limitations: First, most previous studies focused on single dietary patterns rather than comparing multiple dietary patterns, and primarily examined general sleep quality instead of specific sleep phenotypes. Second, systematic decomposition of dietary patterns to identify influential food components remains underdeveloped. Third, while existing studies include cross‐sectional designs, Mendelian randomization analyses, and some prospective cohort or randomized controlled trials, few investigations integrate multiple study designs to triangulate evidence for diet–sleep relationships. This limits the robustness of causal inference. Fourth, current evidence derives predominantly from Western populations, raising concerns about generalizability to other ethnic groups.

To address these gaps, we employed a two‐pronged approach. First, evaluating associations between three dietary pattern indices (MEDI, DASHI, AHEI) and five sleep outcomes (sleep duration, sleep sufficiency, daytime sleepiness, OSA, snoring). Second, decomposing these patterns to examine individual food component contributions across different sleep phenotypes.

Overall, we integrated findings from cross‐sectional analyses, retrospective cohort studies, and Mendelian randomization analyses (MR) to establish causal relationships between diet and sleep outcomes.

## 2 Materials and Methods

### 2.1 Overall Study Design

**FIGURE 1:**
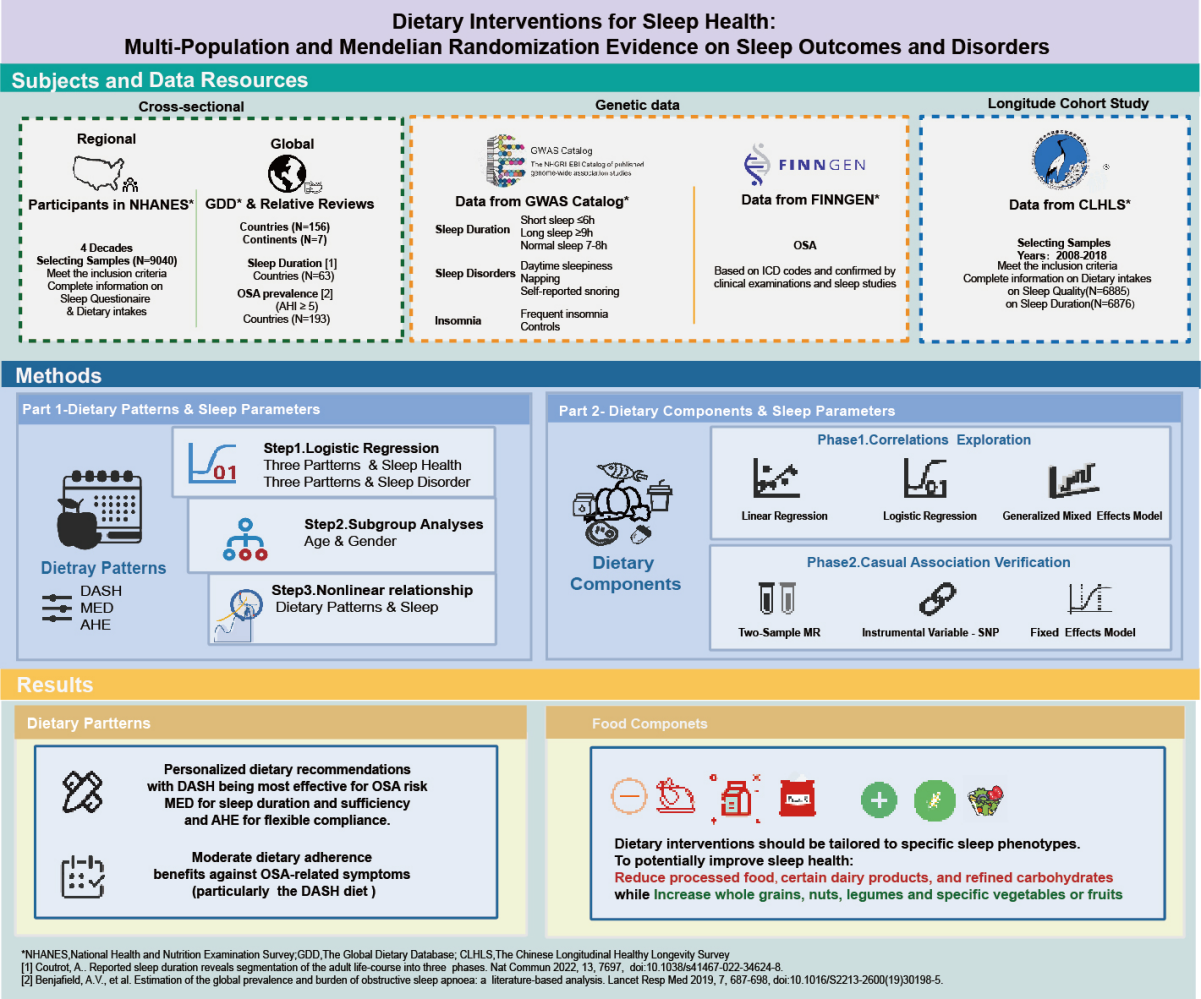
Graphic abstract. This multi‐population study examined three dietary patterns (MED, DASH, AHE) across distinct sleep phenotypes using NHANES data (*n* = 9040), global validation (62 countries), Chinese cohort replication (*n* = 6885), and Mendelian randomization for causal inference. Mediterranean Diet adherence significantly improved sleep duration and sufficiency, while DASH offered the strongest protection against sleep‐disordered breathing. Food‐component analyses revealed consistent benefits from legumes, nuts, and whole grains, with food processing degree critically determining sleep disorder risk. Findings support phenotype‐specific dietary interventions for precision sleep health management.

This study is designed to comprehensively examine the relationships between both dietary patterns and dietary components with sleep outcomes through multiple complementary approaches (Figure 1). For correlation exploration, cross‐sectional analyses using NHANES data examined associations between three dietary patterns and sleep health (duration, sufficiency) and disorders (OSA, daytime sleepiness, snoring, stop‐breathing) in the US population. GDD data explored relationships between dietary components and sleep features across international populations. For validation of cross‐sectional study, we analyzed genome‐wide association study (GWAS) genetic data with MR using genetic variants as instrumental variables to test for causal relationships between dietary components and sleep features, thereby mitigating concerns about reverse causality and confounding. Further validation of sleep health and dietary components utilized CLHLS to assess how dietary patterns predict changes in sleep features over time in an elderly Chinese population.

We adhere to STROBE (Strengthening the Reporting of Observational Studies in Epidemiology) guidelines established for observational study. All analyses were conducted using publicly accessible, anonymized data sets: NHANES, GDD, and CLHLS. As this study involved secondary analysis of de‐identified public data sets, it was exempt from additional institutional review. Ethical permissions obtained during primary data collection covered the present investigation.

### 2.2 Study of Dietary Patterns and Sleep Features

#### 2.2.1 Study Population in NHANES

NHANES data from four cycles (2005–2006, 2007–2008, 2015–2016, 2017–2020) provided detailed dietary patterns via 24‐h dietary recall, sleep outcomes, and relevant covariates including demographics, socio‐economic variables and anthropometric measures. The 2009–2014 cycles were excluded due to the absence of specific sleep disorder variables required for our primary outcomes. Participants with missing covariate data were excluded, yielding a final analytic sample of 9040 participants (Figure S1). The NHANES protocol received approval from the Centers for Disease Control (CDC) and Prevention Research Ethics Review Board.

#### 2.2.2 Exposure Definition

Our investigation employed three validated dietary quality metrics as surrogates for established eating patterns: the index of DASH (DASHI), MED (MEDI), and AHE (AHEI). These indices were derived from mean intakes captured through two consecutive 24‐h dietary recall interviews administered within the NHANES protocol. Implementation code through the R package ‘dietary index’ is publicly accessible at https://github.com/jamesjiadazhan/dietaryindex (Estruch et al. 2013; Kling et al. 2023; Zhan et al. 2024) (Shivappa et al. 2017). We quantified 10 dietary components using standardized definitions to examine their sleep associations. Categories encompassed: plant foods (Whole Grains, Legumes and Nuts, Fruits, Vegetables), animal sources (Red and Processed Meat, Fish/Seafood, Dairy Products), beverages (Alcohol, Sugar‐Sweetened Beverages (SSBs)), and Fats & Oils (Supporting Information S1, Table S1). Self‐reported sleep outcomes validated by previous research included sleep health measures (duration, sufficiency) and sleep disorder symptoms (daytime sleepiness, OSA, snoring frequency, stop‐breathing frequency, insomnia) (Lauderdale et al. 2008). Sleep data were collected via home‐based CAPI interviews and harmonized across NHANES cycles. Sleep duration, measured via SLD010H or derived SLD012, was stratified as: normal (6–9 h), short (< 6 h), or prolonged (> 9 h). Assessments of sleep sufficiency utilized SLQ130 and SLQ050, whereas excessive daytime sleepiness was evaluated through SLQ120. Snoring and breathing issues were coded positive at ≥ 3–4 nights/week via SLQ030 and SLQ040. To align with Healthy People 2030, OSA was defined as meeting any of: physician‐diagnosed sleep apnea (SLQ060), snoring ≥ 3–4 nights/week (SLQ030), snort/gasp/stop‐breathing ≥ 3–4 nights/week (SLQ040), or excessive daytime sleepiness (SLQ120) while reporting ≥ 7 h weekday sleep (from SLD010H/SLD012) (Scinicariello et al. 2017) (Supporting Information S1, Table S2). Multivariable models incorporated: demographic factors (race/ethnicity, marital status, educational attainment, sex, age, poverty‐to‐income ratio), body mass index, behavioral exposures (smoking status, alcohol intake), and prevalent comorbidities (cardiovascular disease, stroke, cancer, liver disease, diabetes, hypertension). Detailed variable definitions and coding are provided in Table S3.

### 2.3 Study of Dietary Components and Sleep Features

#### 2.3.1 Global Dietary Intake

GDD data supplemented dietary component exposures, providing detailed food group intake information across countries. The database captures consumption trends for vegetables, fruits, processed meats, red meats, SSBs, and alcohol. We used median intake data and 95% confidence intervals (95% CI) to evaluate relationships between dietary factors and sleep parameters, particularly OSA. Global reviews contextualized findings through comparative analysis of international trends. Key references included Coutrot's review for global sleep duration across adult life‐course phases (Coutrot et al. 2022), and Benjafield's study for global OSA prevalence and burden estimates highlighting regional disparities (Benjafield et al. 2019). Additional details are provided in Supporting Information S2 and Tables S4, S5.

#### 2.3.2 Mendelian Randomization

We employed MR to assess potential causal links between food preference and sleep phenotypes, leveraging genetic variants as instrumental variables. Dietary preference exposure data were drawn from UK Biobank's comprehensive dietary assessment, which surveyed over 500,000 European‐ancestry participants (May‐Wilson et al. 2022). This expanded questionnaire comprised 152 total items, with 139 specifically capturing food and beverage preferences relevant to our investigation. In total, 183 food liking features were analyzed. Sleep phenotypes included: durational parameters (short/long/total sleep duration) and disorder traits (OSA, insomnia, snoring, daytime napping, excessive daytime sleepiness). OSA data derived from FinnGen biobank (43,901 cases; 366,484 controls). UK Biobank furnished metrics for daytime napping, sleepiness, habitual sleep duration, snoring, and insomnia. Detailed phenotype definitions are presented in Supporting Information S3.

Three quality control steps governed instrument selection: ensuring adequate statistical power (*F* > 10 to minimize weak instrument bias), confirming genetic independence (LD clumping parameters: *r* ^2^ < 0.001 within 10,000‐kb flanking regions), and requiring genome‐wide association evidence (*p* < 5 × 10^−8^) (Palmer et al. 2012). For forward univariate MR analysis, 183 dietary preference traits served as exposures. Initially, SNPs showing genome‐wide associations with sleep outcomes (*p* < 5 × 10^−8^) were removed to mitigate reverse causation. Retained instruments then underwent Steiger directional testing to confirm causal pathways from dietary exposures toward sleep phenotypes.

Our analytical strategy employed inverse variance‐weighted (IVW) estimation as the primary approach, supplemented by multiple sensitivity methods (Burgess et al. 2015). IVW assumes instrument validity but remains susceptible to horizontal pleiotropy. When between‐instrument heterogeneity was detected (Cochran's *Q p* < 0.05), random‐effects IVW was applied; otherwise, fixed‐effects models were used. Robustness verification incorporated four alternative estimators: weighted median (resistant to up to 50% invalid instruments), MR‐Egger regression (detecting directional pleiotropy via intercept assessment), and mode‐based methods (weighted and simple modes). While these techniques relax IVW assumptions, they sacrifice statistical precision. Convergent results across methods strengthen causal inference despite varying accuracy. Diagnostic testing evaluated: heterogeneity through Cochran's *Q* statistic and MR‐PRESSO global assessment; horizontal pleiotropy via MR‐Egger intercept analysis (Hemani et al. 2017).

#### 2.3.3 Cohort Study Design and Population

To complement our MR analysis and address potential limitations of cross‐sectional evidence between diet and sleep health, CLHLS longitudinal analysis addressed cross‐sectional evidence limitations using nationally representative data investigating healthy aging determinants among Chinese older adults (2008–2018). Participants with complete dietary intake, sleep quality, and sleep duration information were included. Dietary exposures were self‐reported on four‐point frequency scales and treated as ordinal variables. Sleep duration (hours/night) and self‐rated sleep quality (5‐point scale) were categorized per National Sleep Foundation guidelines. Eligible participants required complete information on: (1) dietary intakes; (2) sleep quality (*N* = 6885); and (3) sleep duration (*N* = 6876). The study received approval from Peking University Research Ethics Committee (Chen et al. 2024; Zeng 2012) (Supporting Information S4).

### 2.4 Statistical Analysis

Data analyses were executed using R (v4.4.0), Stata (v17.0), and Free Statistics software (v2.1.1). Two‐sided P‐values below 0.05 were considered statistically significant. NHANES data employed pooled survey cycles with CDC‐recommended sampling weights. Details of weight selection and survey design implementation are provided in Supporting Information S1.2.

Continuous variables were compared using *t*‐tests, while categorical variables employed *χ* ^2^ tests. Associations between three dietary indices and five sleep outcomes were estimated via three logistic regression models: Model I presents unadjusted associations showing total effects. Model II adjusted for sociodemographic characteristics (sex, race/ethnicity, education level, marital status, age, income‐to‐poverty ratio) that may confound diet‐sleep associations through cultural, economic, and psychosocial stress pathways. The fully adjusted Model III incorporated: body mass index, lifestyle behaviors (alcohol intake and smoking status), and prevalent comorbidities (diabetes, hypertension, cardiovascular disease, stroke, cancer, liver disease). We treated BMI as a potential intermediate variable, recognizing dietary patterns influence sleep both directly and indirectly through weight modification. Chronic disease adjustments accounted for bidirectional pathways linking comorbidities with both dietary adherence and sleep disturbances. This progressive adjustment clarifies how effect estimates evolve across models. For subgroup analysis, we examined effect modification by sex and age (dichotomized at 60 years) through logistic regression models incorporating interaction terms (diet × sex; diet × age stratum). Interaction effects were tested by including product terms in fully adjusted models. Given multiple hypothesis testing across subgroups, we applied false discovery rate control (FDR) using the Benjamini‐Hochberg method (*q*‐value < 0.05). *P*‐values for interaction terms are reported for each dietary pattern‐sleep outcome combination. Potential nonlinear exposure‐response patterns were modeled using restricted cubic spline frameworks (RCS).

Global dietary exposures from GDD were assessed via generalized linear mixed models (GLMMs) to accommodate participant clustering within countries nested under continents. Negative binomial distribution (log link) was used for Apnea Hypopnea Index (AHI) counts, and Gaussian distribution (log link) for sleep duration. Model selection employed Bayesian Information Criterion (BIC) and Akaike Information Criterion (AIC) (Miller et al. 2022).

CLHLS utilized fixed‐effects regression assessing within‐person associations between dietary frequency and sleep outcomes over 2008–2018, adjusting for time‐varying covariates: age, health indicators (BMI), functional capacity (activities of daily living, cognitive performance, and physical activity), socioeconomic factors (economic status, marital status). Robust clustered standard errors accounted for serial correlation and heteroscedasticity (Wang et al. 2023; Wang and Yang 2022). Further details are provided in the Supporting Information S5.

## 3 Results

### 3.1 Dietary Pattern and Sleep Features

#### 3.1.1 Participants Characteristics Stratified by OSA in NHANES

The study included 9040 participants, with a mean age of 48 years. The sample was composed of 52.01% females and 47.99% males. 42.51% are non‐Hispanic White population. Regarding educational attainment, 55.12% of participants had completed some college or higher. In terms of marital status, 62.31% of participants were living with a partner. Furthermore, 55.07% had never smoked. Self‐reported OSA was present in 49.24% of participants, including snoring occasionally or more often (54.18%), snorting or breathing interruptions (11.61%), and excessive daytime sleepiness despite adequate sleep (22.21%). Additionally, 26.75% reported insufficient sleep. The prevalence of chronic diseases varied significantly between the OSA and non‐OSA groups, as presented in Table 1.

**TABLE 1:** Characteristics of NHANES participants by self‐reported OSA result.

Characteristics	Total	Self‐reported OSA ‐negative	Self‐reported OSA ‐positive	Weighted p*
*N*		4589 (50.76%)	4451 (49.24%)	
Age (years)	48.09 ± 0.39	46.49 ± 0.46	49.74 ± 0.41	< 0.001
Age Level				0.993
< 60	6102 (67.50%)	3127 (68.14%)	2975 (66.84%)	
> 60	2938 (32.50%)	1462 (31.86%)	1476 (33.16%)	
Gender (%)				< 0.001
Male	4338 (47.99%)	1931 (42.08%)	2407 (54.08%)	
Female	4702 (52.01%)	2658 (57.92%)	2044 (45.92%)	
Race (%)				0.98
Mexican American	1412 (15.62%)	688 (14.99%)	724 (16.27%)	
Other Hispanic	846 (9.36%)	395 (8.61%)	451 (10.13%)	
Non‐Hispanic White	3843 (42.51%)	1991 (43.39%)	1852 (41.61%)	
Non‐Hispanic Black	2053 (22.71%)	1041 (22.68%)	1012 (22.74%)	
Other Race‐Including Multi‐Racial	886 (9.80%)	474 (10.33%)	412 (9.26%)	
Education (%)				< 0.001
Less than high school	1959 (21.67%)	945 (20.59%)	1014 (22.78%)	
High school	2098 (23.21%)	1012 (22.05%)	1086 (24.40%)	
Some college	2879 (31.85%)	1504 (32.77%)	1375 (30.89%)	
Bachelors' degree or higher	2104 (23.27%)	1128 (24.58%)	976 (21.93%)	
Marital status (%)				< 0.001
Married/living with Partner	5633 (62.31%)	2632 (57.35%)	3001 (67.42%)	
Widowed/divorced/separated	2338 (25.86%)	1335 (29.09%)	1003 (22.53%)	
Never married	889 (9.83%)	536 (11.68%)	353 (7.93%)	
Separated	180 (1.99%)	86 (1.87%)	94 (2.11%)	
Family income poverty ratio	3.09 ± 0.04	3.1 ± 0.04	3.09 ± 0.05	0.897
BMI	29.13 ± 0.11	27.53 ± 0.17	30.79 ± 0.15	< 0.001
Smoking (%)				< 0.001
Never	4978 (55.07%)	2713 (59.12%)	2265 (50.89%)	
Former	1753 (19.39%)	808 (17.61%)	945 (21.23%)	
Current	2309 (25.54%)	1068 (23.27%)	1241 (27.88%)	
Liver disease (%)				0.126
No	8642 (95.60%)	4418 (96.27%)	4224 (94.90%)	
Yes	398 (4.40%)	171 (3.73%)	227 (5.10%)	
Stroke (%)				0.329
No	8657 (95.76%)	4419 (96.30%)	4238 (95.21%)	
Yes	383 (4.24%)	170 (3.70%)	213 (4.79%)	
Cancer (%)				0.1
No	8164 (90.31%)	4160 (90.65%)	4004 (89.96%)	
Yes	876 (9.69%)	429 (9.35%)	447 (10.04%)	
CVD (%)				0.033
No	8223 (90.96%)	4207 (91.68%)	4016 (90.23%)	
Yes	817 (9.04%)	382 (8.32%)	435 (9.77%)	
Hypertension (%)				< 0.001
No	6337 (70.10%)	3405 (74.20%)	2932 (65.87%)	
Yes	2703 (29.90%)	1184 (25.80%)	1519 (34.13%)	
Diabetes (%)				< 0.001
No	7608 (84.16%)	4006 (87.30%)	3602 (80.93%)	
Yes	1432 (15.84%)	583 (12.70%)	849 (19.07%)	
Sleep_hours	7.26 ± 0.02	7.34 ± 0.03	7.17 ± 0.03	< 0.001
Sleep_Sufficiency				< 0.001
Insufficient (< 6 h)	2418 (26.75%)	1127 (24.56%)	1291 (29.00%)	
Adequate (6–9 h)	4765 (52.71%)	2458 (53.56%)	2307 (51.83%)	
Sufficient (> 9 h)	1857 (20.54%)	1004 (21.88%)	853 (19.16%)	
Snor (%)				< 0.001
Never	2233 (24.70%)	2119 (46.18%)	114 (2.56%)	
Rarely	1909 (21.12%)	1798 (39.18%)	111 (2.49%)	
Occasionally	1603 (17.73%)	0 (0.00%)	1603 (36.01%)	
Frequently	3295 (36.45%)	672 (14.64%)	2623 (58.93%)	
Snort & stop breathing (%)				< 0.001
Never	7022 (77.68%)	4154 (90.52%)	2868 (64.43%)	
Rarely	968 (10.71%)	435 (9.48%)	533 (11.97%)	
Occasionally	549 (6.07%)	0 (0.00%)	549 (12.33%)	
Frequently	501 (5.54%)	0 (0.00%)	501 (11.26%)	
Sleepy (%)				< 0.001
Never	2203 (24.37%)	1217 (26.52%)	986 (22.15%)	
Rarely	2052 (22.70%)	1169 (25.47%)	883 (19.84%)	
Sometimes	2778 (30.73%)	1390 (30.29%)	1388 (31.18%)	
Often	1361 (15.06%)	660 (14.38%)	701 (15.75%)	
Almost always	646 (7.15%)	153 (3.33%)	493 (11.08%)	

*Note:* All estimates incorporated survey weights to represent the US civilian, noninstitutionalized population. “*” denotes a weighted statistical value, as indicated by “Weighted p*”. Continuous measures are expressed as mean ± standard error; categorical measures as weighted frequency (percentage).

Abbreviations: BMI, body mass index; CVD, cardiovascular disease; OSA, obstructive sleep apnea; SSB, sugar‐sweetened beverage.

#### 3.1.2 Logistics Analysis

**FIGURE 2:**
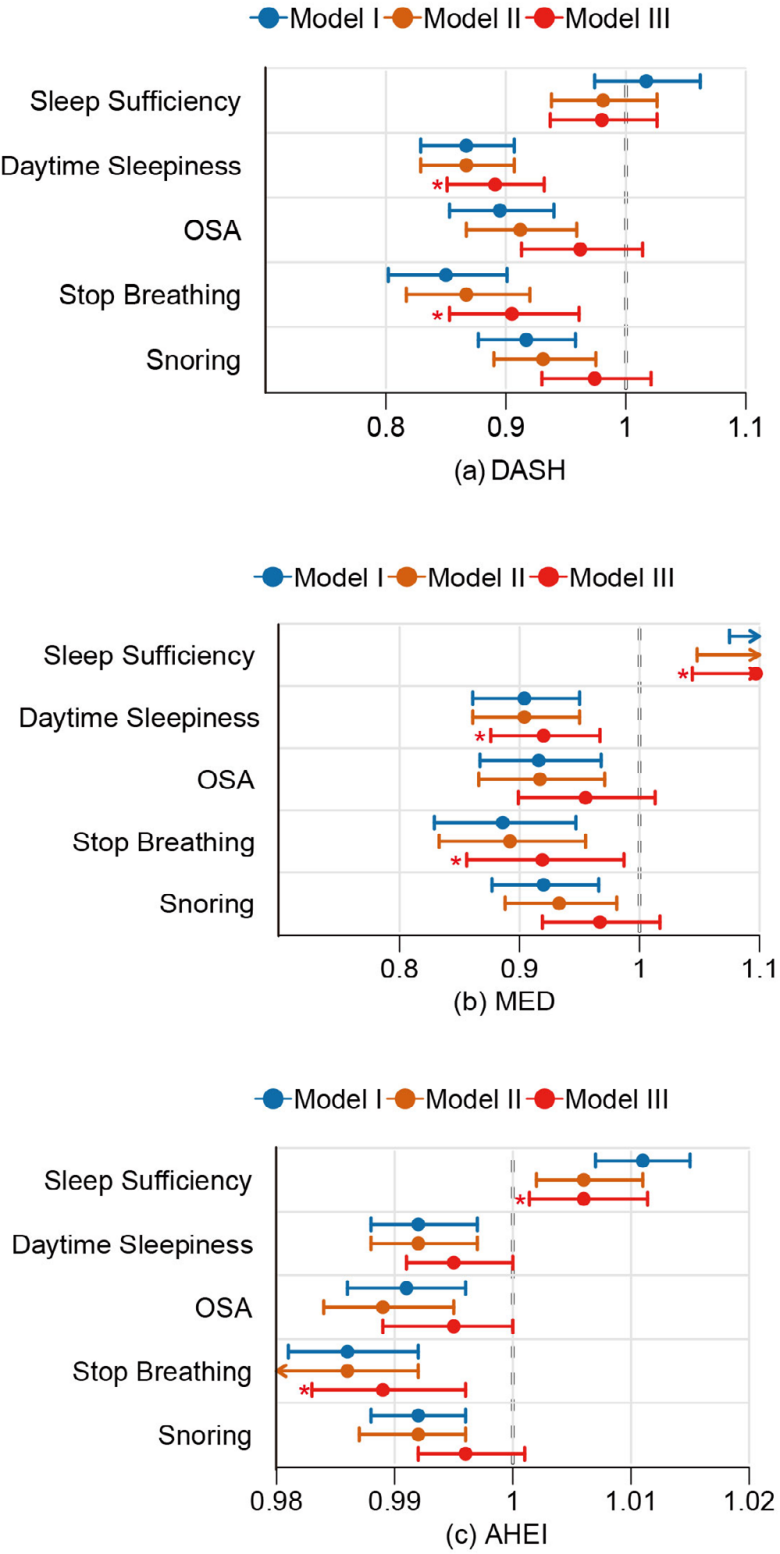
Weighted Logistic Models Evaluating Associations Between Dietary Patterns and Sleep Features. “*” indicates a *p*‐value in Model III < 0.001. Model I, Crude Model. Model II, Adjusted for six sociodemographic variables: Race/ethnicity, marital status, educational attainment, sex, age, and income‐to‐poverty ratio. Model III, Extended adjustment adding BMI, smoking status, and comorbidity history (hypertension, diabetes, cardiovascular disease, cerebrovascular events, malignancies, hepatic conditions) to Model II covariates.

Figure 2 presents the associations between three dietary patterns and sleep outcomes across adjustment models. In the fully adjusted Model III, the three dietary patterns demonstrated distinct associations with sleep features. The DASH dietary pattern showed significant associations with two sleep features: daytime sleepiness (OR = 0.89, 95% CI: 0.85–0.93, *p* < 0.001) and breathing cessation episodes (OR = 0.91, 95% CI: 0.85–0.96, *p* = 0.001). Notably, while snoring and OSA exhibited significant associations in Models I and II, these relationships were attenuated to non‐significance following full adjustment (*p* > 0.05). The MED demonstrated the most comprehensive beneficial associations in Model III: sleep sufficiency (OR = 1.10, 95% CI: 1.04–1.15, *p* < 0.001), daytime sleepiness (OR = 0.92, 95% CI: 0.88–0.97, *p* = 0.001), and stop breathing (OR = 0.92, 95% CI: 0.86–0.99, *p* = 0.02). These associations remained robust across all adjustment levels. However, no significant associations were observed for snoring or OSA in Model III, despite these features showing significant associations in less adjusted models. The AHE dietary pattern‐maintained associations with sleep sufficiency (OR = 1.00, 95% CI: 1.00–1.01, *p* = 0.015), daytime sleepiness (OR = 0.99, 95% CI: 0.99–0.99, *p* = 0.046), and stop breathing (OR = 0.99, 95% CI: 0.98–0.99, *p* = 0.062) in Model III. Further details are provided in the Supporting Information S7, Table S8.

#### 3.1.3 Subgroup Analysis

**FIGURE 3:**
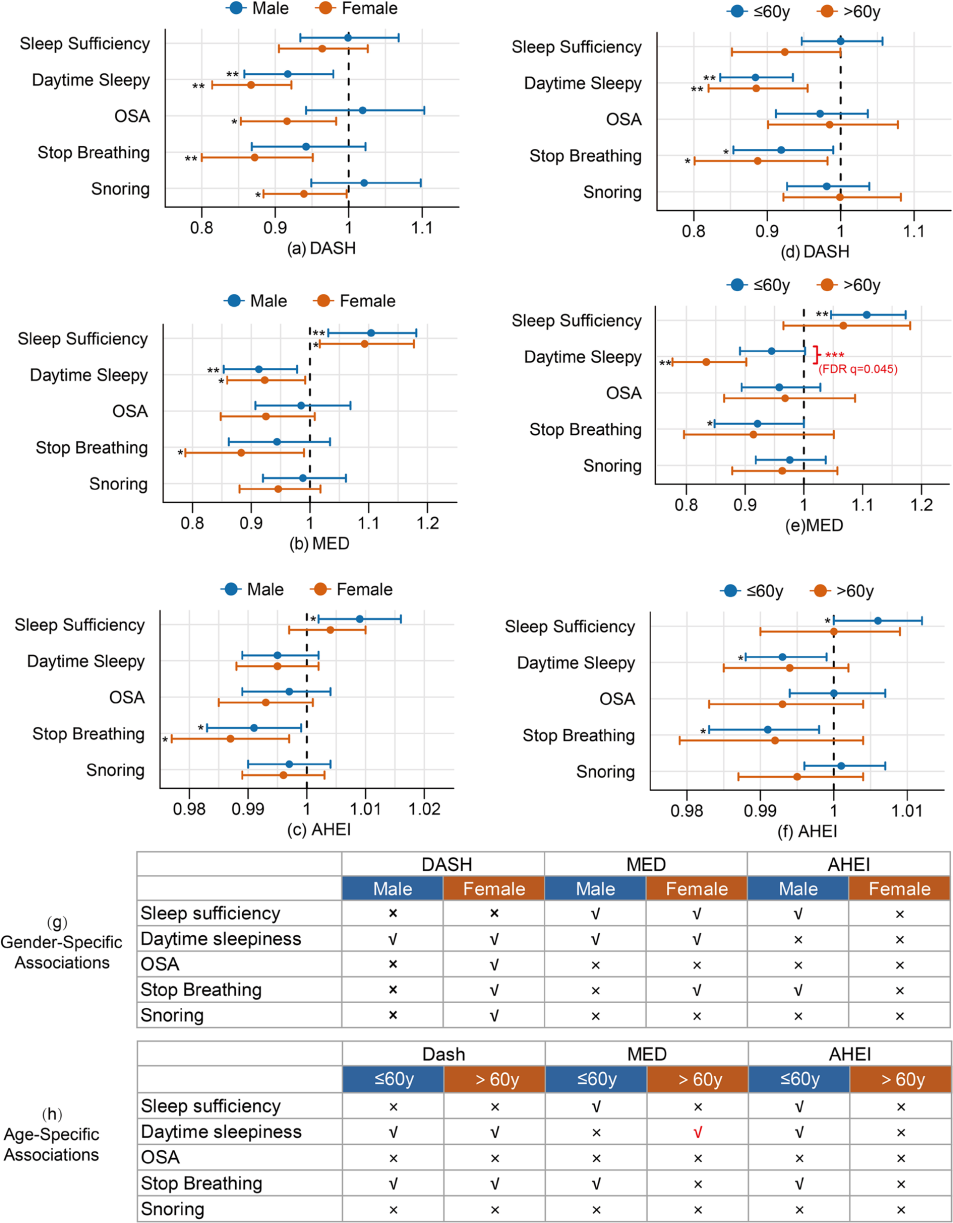
Subgroup Effects of Three Dietary Patterns (MED, DASH, AHEI) on Sleep Metrics by Sex and Age. Red highlights indicate statistically significant associations with FDR *q* < 0.05. (a–c) represent the subgroup analysis by gender; (d–f) represent the subgroup analysis by age. “*” represents *p* < 0.05; “**”represents *p* < 0.01; “***” represents the corresponding p for interaction between groups. (g–h) represent the gender or age specific associations, summary of directional trends (“√” represents *p* < 0.05; “×” represents *p* ≥ 0.05).

We performed stratified analyses by sex and age groups (≤ 60 vs. > 60 years) with formal interaction testing. The analysis of gender‐specific interactions in Figure 3a–c revealed minimal effect modification by gender for most dietary pattern‐sleep outcome relationships (all FDR.*p* > 0.05). Gender stratification revealed some potential benefits among females, DASH dietary adherence was significantly associated with reduced OSA (OR = 0.92, 95% CI: 0.85–0.98, *p* = 0.015), stop breathing (OR = 0.87, 95% CI: 0.80–0.95, *p* = 0.002), and snoring (OR = 0.94, 95% CI: 0.88–0.99, *p* = 0.040). Age stratification in Figure 3d–f demonstrated more pronounced heterogeneity, with MED showing significant effect modification for sleep sufficiency (FDR.*p* = 0.045). The MED pattern exhibited age‐dependent effects on daytime sleepiness (*p* for interaction = 0.009), with substantially stronger protective associations in adults > 60 years (OR = 0.83, 95% CI: 0.76–0.90, *p* < 0.001) compared to younger individuals (OR = 0.95, 95% CI: 0.89–1.00, *p* = 0.059). DASH pattern showed some difference in improving younger adults' sleep sufficiency. For the AHE pattern, beneficial associations were generally more pronounced in younger adults (≤ 60 years), with relative improvements in sleep sufficiency, daytime sleepiness, and stop breathing episodes (Supporting Information S7, Table S9). A comprehensive overview of effect estimate distributions across gender and age subgroups is provided in Figure 3g–h.

#### 3.1.4 Restricted Cubic Spline Analyses

**FIGURE 4:**
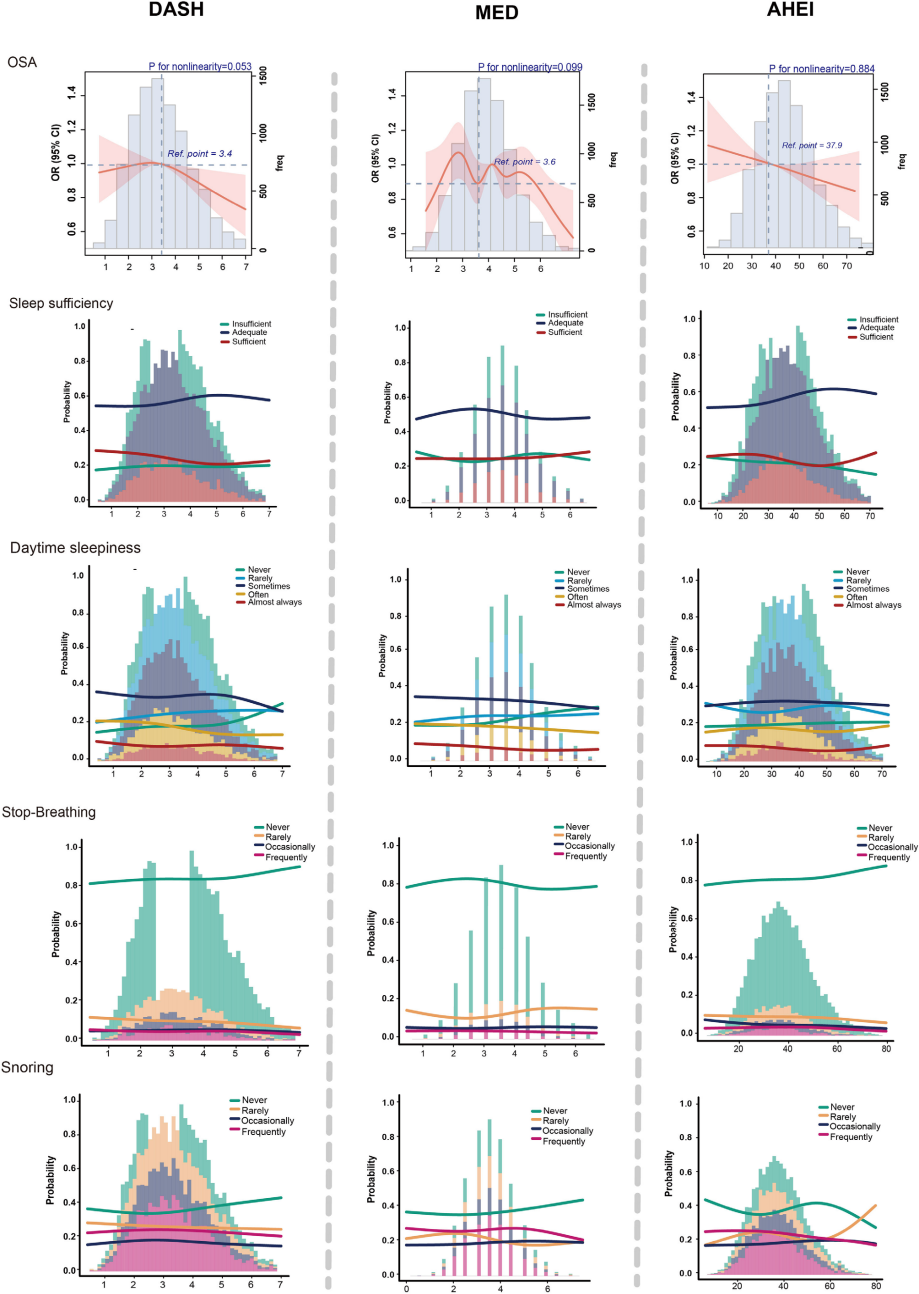
Restricted Cubic Spline Curves Illustrating Associations Between Dietary Pattern Scores and Sleep Features. (1) Histograms in the background show the distribution of scores for each dietary pattern. (2) The *x*‐axis represents dietary pattern scores (ranging from approximately 1–7 for DASH and Mediterranean diets, and 10–70 for AHEI). (3) The *y*‐axis shows the estimated odds ratios (95% CI) or probability of each response category. OSA: The solid red lines represent the 95% CI for OSA risk across different dietary pattern scores. Shaded areas represent the 95% confidence intervals. Others: Multicolored curves represent the probability of different response categories across dietary pattern scores.

RCS analysis revealed quantified dose–response relationships between dietary scores and sleep symptom risks that increasing dietary adherence was consistently associated with lower frequency of sleep symptoms (Figure 4). OSA risk demonstrated notable inflection points: DASH diet benefits emerged at scores ≥ 3.4, AHEI at ≥ 37.9, while MED exhibited an “M‐shaped” curve with initial risk reduction at moderate adherence (score ≈3.6) and sustained benefits beyond 5.5. These findings indicate that even moderate dietary improvements confer substantial sleep‐related benefits, with additional incremental advantages at higher adherence levels.

### 3.2 Dietary Components and Sleep Features

#### 3.2.1 Correlation Analysis Between Dietary Components and Sleep Features in NHANES

**FIGURE 5:**
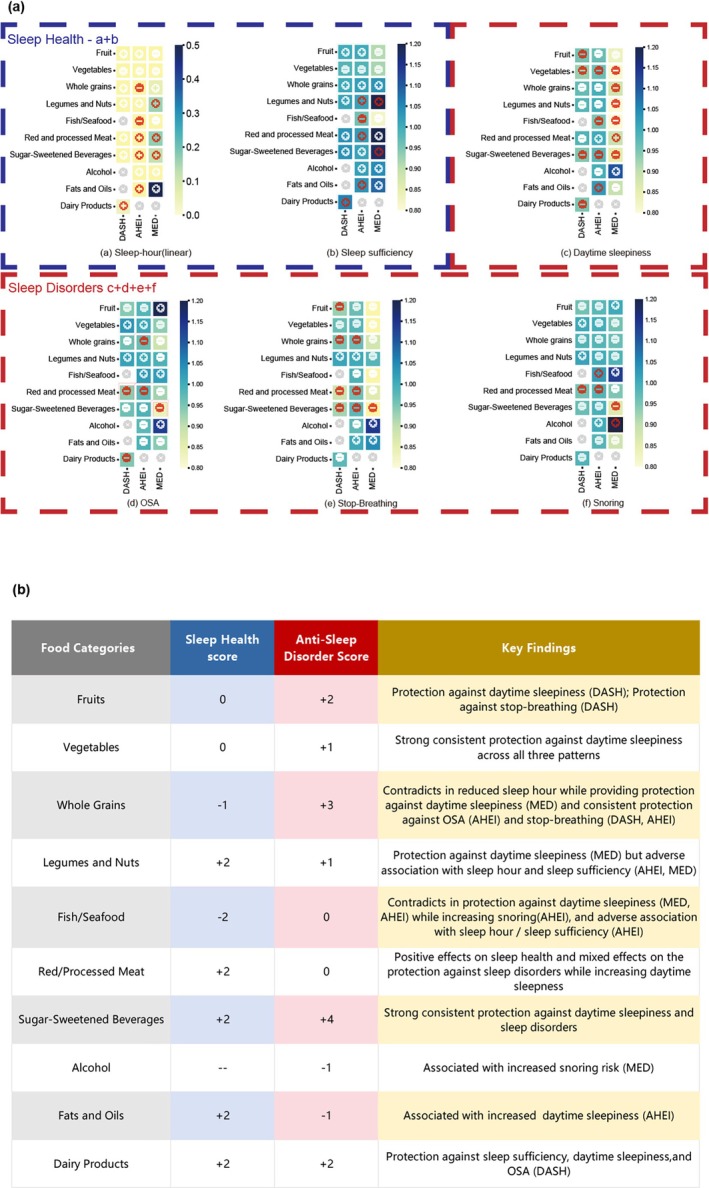
Heatmap Analysis and Scoring Summary of Dietary Categories Associations with Sleep Features Across Dietary Patterns. (a) This heatmap displays associations between 10 dietary categories across three dietary patterns (DASH, MED, AHEI) and sleep parameters. The Blue border shows sleep health metrics (sleep duration, sufficiency), while the Red border presents sleep disorder indicators (OSA, stop‐breathing, snoring, daytime sleepiness). The color scales on the right of each figure represent *β* coefficients for sleep time and odds ratios (OR) for all other sleep theater. In every small box, filled red circles represent statistically significant associations (*p* < 0.05). Gray circles indicate non‐significant findings. “+” shows protective relationships and “−” shows detrimental ones. (b) provides an enhanced summary table that quantifies the scores for dietary categories' associations with sleep health and anti‐sleep disorders. Scores are derived based on statistically significant associations from the heatmap (a) The details of the scoring system are provided in Supporting Information S6 and S7, Table S10.

We performed correlation analysis on dietary components from three dietary patterns (DASH, MED, and AHE) and sleep symptoms using data from 9040 NHANES participants. The analysis was visualized through heatmaps (Figure 5a), with scoring systems detailed in Figure 5b and Supporting Information S6.

Regarding sleep duration, several dietary components were associated with extended sleep time, including legumes/nuts, red/processed meat, SSBs, dairy products, and fats/oils. Conversely, whole grains and fish/seafood correlated with shortened sleep duration, while fruits and vegetables showed no significant associations.

For sleep disorder improvement, plant‐based food groups—fruits, vegetables, whole grains, and legumes/nuts—consistently demonstrated beneficial effects. Dairy products and SSBs also showed positive associations with OSA improvement. However, alcohol consumption and fats/oils were associated with detrimental effects on OSA symptoms, while red/processed meat showed inconsistent relationships across different symptoms.

These bidirectional relationships in NHANES highlight the complex nature of diet‐sleep interactions, suggesting that dietary interventions for sleep improvement require tailored approaches specific to individual sleep concerns rather than universal recommendations.

#### 3.2.2 Correlation Analysis Between Dietary Components and Sleep Features in GDD

**FIGURE 6:**
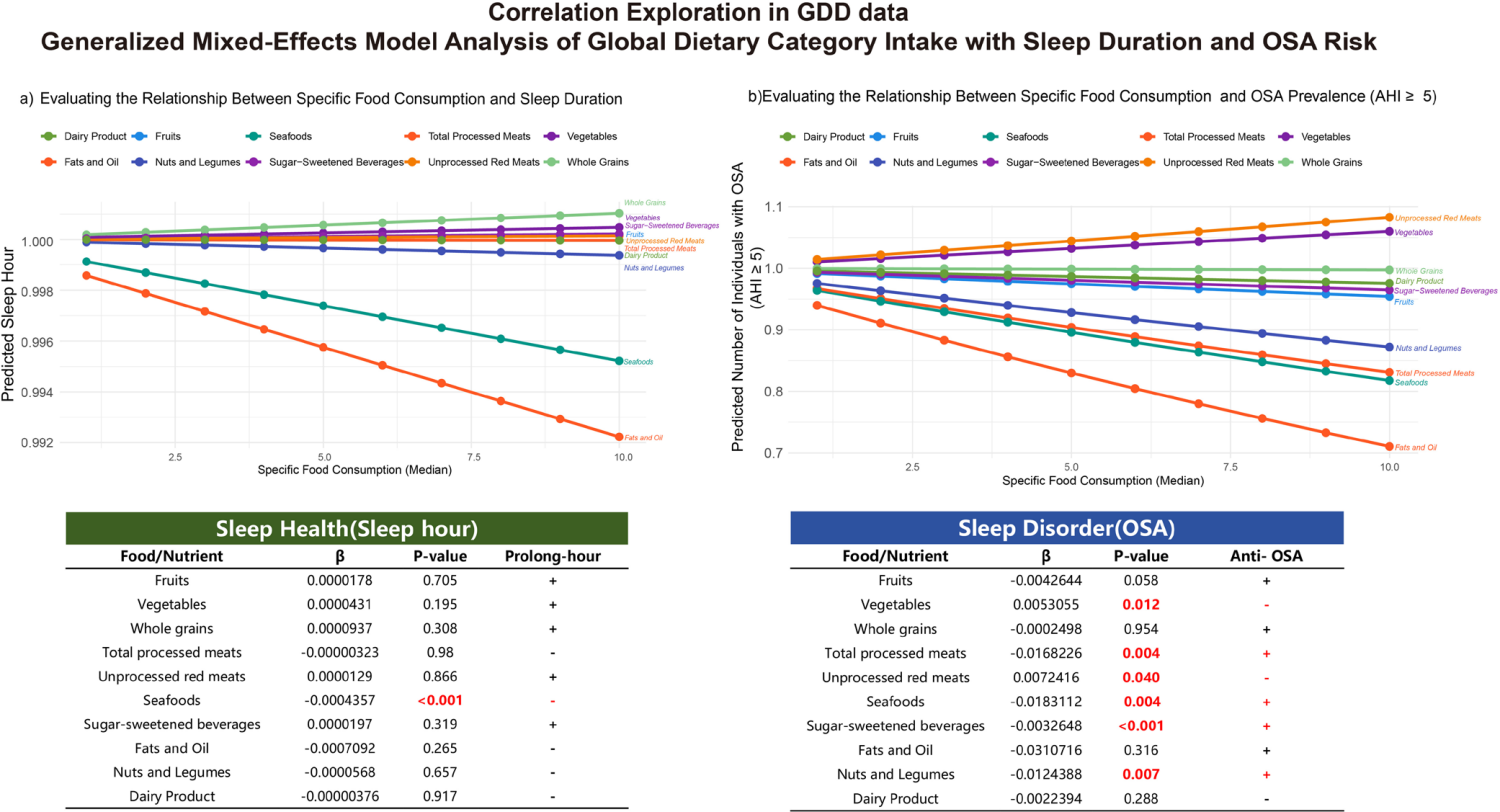
Generalized Mixed‐Effects Model Analysis of Global Dietary Components Intake with Sleep Features and OSA Risk. (a) Specific Food Consumption and Sleep Duration. (b) Specific Food Consumption and OSA Prevalence (AHI ≥ 5). The details are provided in Supporting Information S7, Tables S11 and S12.

We examined global associations between dietary components and sleep features by integrating dietary intake data from the Global Dietary Database (GDD) with sleep health outcomes across 62 countries and OSA prevalence data across 191 countries. A generalized mixed‐effects model was applied to assess these relationships while adjusting for regional, demographic, and socioeconomic variations (Figure 6).

Limited significant associations emerged between dietary components and sleep duration. Among all components examined, only seafood consumption demonstrated a statistically significant negative correlation with sleep duration.

Multiple significant associations were observed between dietary components and OSA prevalence (AHI ≥ 5). Processed meats, seafood, SSBs, and legumes/nuts showed significant negative associations with OSA risk, indicating potential protective effects. Conversely, vegetables and unprocessed red meat showed significant positive associations with OSA risk.

The divergent findings regarding unprocessed versus processed meat warrant attention, with unprocessed red meat correlating with increased OSA risk while processed meat appeared protective against OSA.

#### 3.2.3 Causality Exploration Between Dietary Components and Sleep Features With MR

The MR analysis identified some significant causal associations between food preferences and sleep outcomes (FDR‐corrected *p* < 0.05). Due to the involvement of multiple tests, we applied FDR correction, and results with an FDR‐corrected *p*‐value < 0.05 were considered statistically significant (Supporting Information S3, Table S7). Raw *p*‐values < 0.05 were considered potential positive results.

**FIGURE 7:**
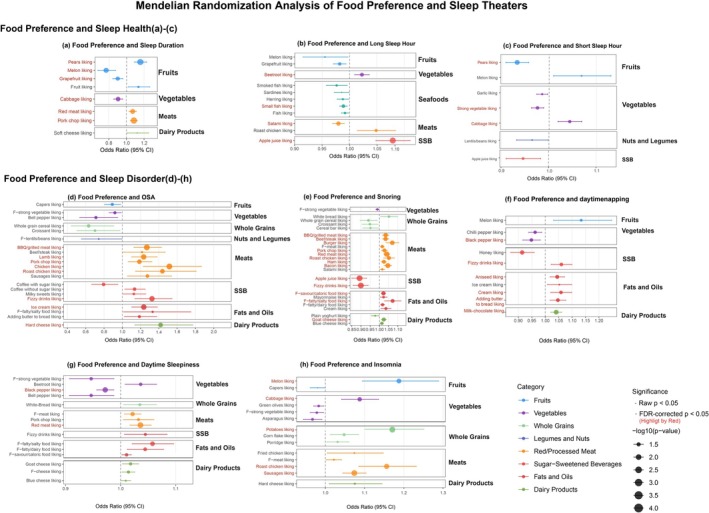
Mendelian Randomization Analysis of Food Preference and Sleep Features. (a–c) Food Preference and Sleep Duration: (a) Sleep Duration (b) Short Sleep Hour (c) Long Sleep Hour. (d–h) Food Preference and Sleep Duration: (d) OSA (e) Snoring (f) Daytime Napping (g) Daytime Sleepiness (h) Insomnia. The details are provided in Supporting Information S3, Tables S6 and S7.

Significant associations were observed between food‐liking traits and sleep duration after FDR correction. Cabbage, grapefruit, and melon were negatively associated with sleep duration (ORs: 0.91 [95% CI: 0.86–0.96], 0.90 [95% CI: 0.84–0.96], and 0.76 [95% CI: 0.67–0.87], respectively), while pears, pork chop, and red meat were positively associated (ORs: 1.16 [95% CI: 1.09–1.23], 1.09 [95% CI: 1.05–1.12], and 1.07 [95% CI: 1.03–1.11], respectively) (Figure 7a). Beetroot and apple juice liking were positively associated with long sleep duration (ORs: 1.02 [95% CI: 1.01–1.04] and 1.08 [95% CI: 1.05–1.11]), while salami and the small fish liking factor were negatively associated (ORs: 0.98 [95% CI: 0.97–0.99] and 0.99 [95% CI: 0.98–1.00]) (Figure 7b). Cabbage liking was positively associated with short sleep (OR = 1.04 [95% CI: 1.02–1.07]), while pears and the strong vegetable factor were negatively associated (ORs: 0.93 [95% CI: 0.91–0.96] and 0.98 [95% CI: 0.97–0.99]) (Figure 7c).

For sleep disorders, BBQ/grilled meat, diet fizzy drinks, hard cheese, ice cream, lamb, and pork chop were positively associated with OSA (ORs: 1.27 [95% CI: 1.13–1.43], 1.32 [95% CI: 1.14–1.54], 1.42 [95% CI: 1.14–1.77], 1.23 [95% CI: 1.11–1.37], 1.23 [95% CI: 1.12–1.36], and 1.19 [95% CI: 1.09–1.30], respectively). Chicken and roast chicken also showed positive associations with OSA risk (ORs: 1.51 [95% CI: 1.23–1.87] and 1.44 [95% CI: 1.14–1.81]) (Figure 7d).

Snoring risk was positively associated with liking bacon, BBQ/grilled meat, beef/steak, goat cheese, ham, pork chop, red meat, and roast chicken (ORs: 1.02 to 1.09). The fatty/salty and savory/caloric food factors also showed positive associations (ORs: 1.09 [95% CI: 1.05–1.13] and 1.02 [95% CI: 1.01–1.03]). In contrast, apple juice and fizzy drinks were associated with reduced snoring risk (ORs: 0.89 [95% CI: 0.85–0.93] and 0.90 [95% CI: 0.86–0.94]), while burger liking increased the risk (OR = 1.07 [95% CI: 1.03–1.11]) (Figure 7e).

Food preferences for butter on bread, aniseed, cream, milk chocolate, and fizzy drinks were positively associated with daytime napping (ORs: 1.04 to 1.12), whereas black pepper liking was inversely associated with both daytime napping (OR = 0.94 [95% CI: 0.92–0.96]) and daytime sleepiness (OR = 0.97 [95% CI: 0.96–0.99]). Red meat liking was positively associated with daytime sleepiness (OR = 1.04 [95% CI: 1.02–1.06]) (Figure 7f,g).

For insomnia, cabbage and sausages were positively associated (ORs: 1.09 [95% CI: 1.05–1.12] and 1.07 [95% CI: 1.04–1.10]). Melon, potatoes, and roast chicken were also associated with an increased risk of insomnia (ORs: 1.19 [95% CI: 1.09–1.29], 1.17 [95% CI: 1.10–1.25], and 1.16 [95% CI: 1.09–1.23]) (Figure 7h).

Across all results above, the MR‐Egger intercept indicated no evidence of horizontal pleiotropy. The Steiger test, which evaluates whether the direction of causality is in line with the hypothesized exposure–outcome relationship, showed a *p*‐value far smaller than 0.05. Furthermore, most of sensitivity analyses using methods other than IVW and Wald ratio, such as weighted median and MR Egger, showed consistent directions of effect. Only a small proportion of estimates exhibited heterogeneity. Detailed results are provided in Table S7a,b.

#### 3.2.4 Cohort Perspective From CLHLS Between Dietary Components and Sleep Duration

To address cross‐sectional study limitations, we conducted longitudinal analysis using fixed‐effects models with CLHLS data from 2008 to 2018. This approach complements our MR findings by examining how changes in dietary patterns within individuals over time relate to changes in sleep outcomes.

**FIGURE 8:**
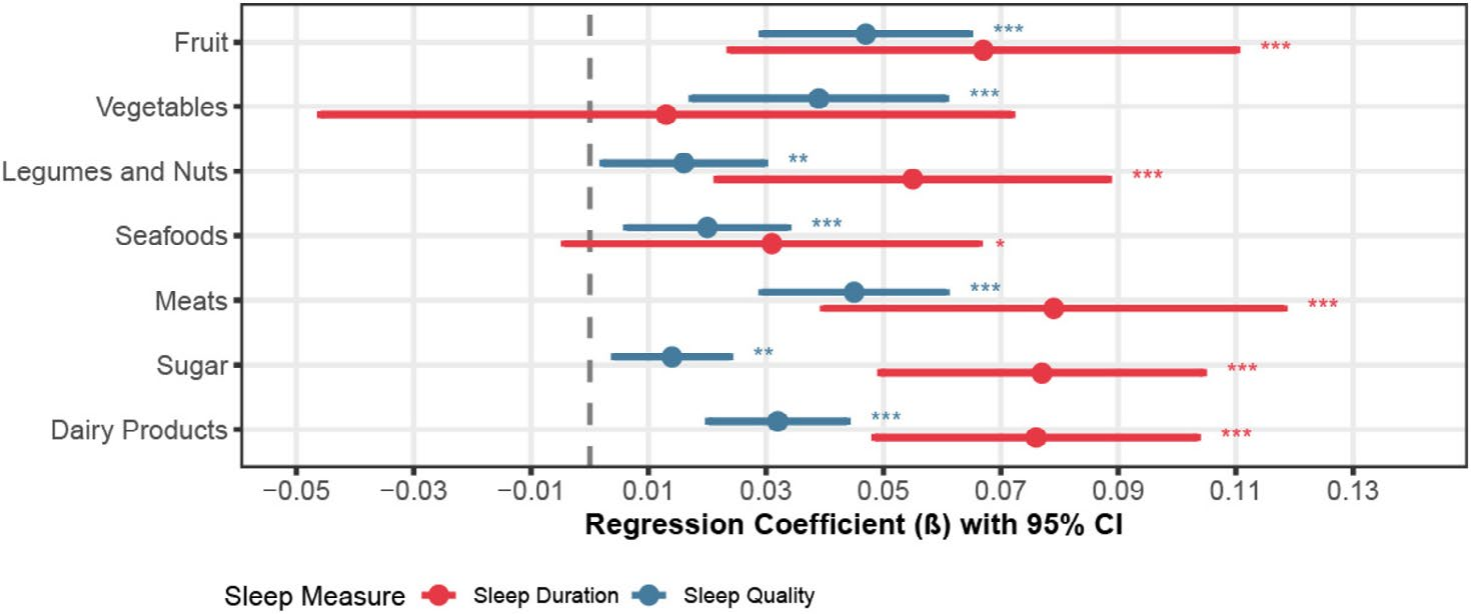
Association Between Dietary Factors and Sleep Health. *p < 0.1, **p < 0.05, ***p < 0.01. Covariables: Age, economic status, marital status, BMI, exercise, activities of daily living, cognitive function, and time effects. All models used fixed effects (FE) estimation with robust standard errors. The details are provided in Supporting Information S7, Table S13.

Fixed‐effects models revealed predominantly positive associations between dietary factors and both sleep quality and duration (Figure 8). Plant‐based foods showed consistent benefits: fruit consumption demonstrated significant positive associations with both sleep quality (*β* = 0.067, *p* < 0.01) and duration, while vegetable consumption significantly improved sleep quality (*β* = 0.039, *p* < 0.01). Garlic consumption was positively associated with both outcomes (quality: *β* = 0.017, *p* < 0.01; duration: *β* = 0.050, *p* < 0.01), supporting our MR findings.

Among protein sources, meat consumption showed strong positive associations with both sleep quality (*β* = 0.045, *p* < 0.01) and duration (*β* = 0.079, *p* < 0.01). Fish and beans demonstrated similar beneficial effects (*β* = 0.020–0.085, *p* < 0.01). Dairy products were strongly associated with both outcomes, while nuts showed stronger associations with sleep duration (*β* = 0.055, *p* < 0.01) than quality.

These cohort findings provide stable temporal evidence supporting beneficial effects of diverse dietary components on sleep health (Supporting Information S7, Table S13).

## 4 Discussion

In this study, using multinational data sets, we examined multiple dietary patterns and specific food across various sleep phenotypes. Our analysis revealed two key findings. First, we found that adherence to MED, DASH, and AHEI dietary patterns was associated with improved sleep outcomes. Second, our findings revealed distinct food effects on sleep outcomes. Legumes, nuts, red meat, SSBs, and dairy extended sleep duration. Legumes, nuts, and whole grains protected against sleep disorders, while fruits, vegetables, meats, dairy, and beverages showed mixed effects. Food processing degree critically determined sleep disorder risk.

Our study also presents two key innovations. Although dietary patterns show associations with sleep outcomes across previous studies, our study uniquely compares multiple dietary approaches within the same population, enabling direct evaluation of relative effectiveness across diverse sleep phenotypes. We examined sleep from two complementary perspectives: sleep duration (physiological condition) and sleep disorders (pathological condition) which provide potential dietary guidance for different sleep presentations in clinical practice. Additionally, we integrated multinational data sources including NHANES and GDD through cross‐sectional analyses, CLHLS data for cohort investigations, and GWAS, substantially enhancing external validity and generalizability of our findings across diverse global populations.

Our findings are consistent with related research (Godos et al. 2025), demonstrating that dietary patterns exhibit distinct efficacy profiles across sleep features. The DASH pattern may be associated with benefits for OSA symptoms and daytime sleepiness, while the MED pattern showed associations with sleep duration and sufficiency concerns and AHEI's flexible framework offers balanced impacts across multiple sleep features and superior long‐term adherence potential.

The results also showed age‐specific and gender‐differential associations with dose–response patterns. Subgroup analyses revealed limited effect modification after FDR correction. After FDR correction, only the MED × age interaction for daytime sleepiness survived multiple‐testing adjustment (FDR *q* = 0.045), with older adults showing markedly greater benefit. This age‐specificity may hypothetically reflect converging physiological changes according to former researchers: elevated inflammatory burden and oxidative damage in aging populations potentially increasing responsiveness to MED's antioxidants (polyphenols, omega‐3s) (Campanini et al. 2017; Golmohammadi et al. 2025; Jaussent et al. 2011). Regarding sex stratification, while no interactions survived FDR correction, descriptive patterns suggested potential female‐predominant associations for certain outcomes. Women may experience greater benefits from protective dietary patterns for OSA‐related symptoms. However, the associations require investigation in further studies.

RCS analyses identified potential dose–response patterns. Linear dose–response relationships confirm that incremental dietary improvements translate into measurable sleep benefits. This finding underscores the utility of emphasizing even small, sustainable changes in eating patterns—such as adding one extra serving of fruits and vegetables per day or reducing processed food intake—as tangible, early targets in patient counseling. Critical inflection points for OSA risk were identified: MED pattern benefits emerged at moderate adherence (score ≈3.6) with sustained protection beyond 5.5. These observations may inform future clinical research on therapeutic thresholds and support precision nutrition for sleep disorders.

The robust associations between dietary patterns and sleep features in NHANES suggest a novel paradigm for personalized nutritional interventions tailored to individual sleep phenotype (Potts et al. 2023). However, dietary pattern studies cannot clarify which specific food components drive these associations. We therefore analyzed associations between specific food components and sleep outcomes to understand the underlying biological basis. Previous systematic reviews identify three primary pathways through which dietary components influence sleep: The first pathway involves neurotransmitter synthesis, particularly serotonin and melatonin production (Golmohammadi et al. 2024). The second pathway operates through inflammatory response modulation, including systemic inflammation and oxidative stress (Golmohammadi et al. 2024). The third pathway encompasses metabolic‐mechanical effects, including glycemic control, adiposity regulation, and upper airway patency changes via fluid retention (St‐Onge et al. 2016). Therefore, different food types mainly converge on these three mechanisms to influence sleep through distinct biological pathways. We use these to help explain the biological basis of our findings.

Our multi‐database analysis integrating NHANES, GDD, MR analyses, and CLHLS cohort data examines associations between specific food components and sleep disorders. Multi‐database evidence supported positive associations between legumes/nuts, red meat, SSBs, and dairy products with extended sleep duration. For sleep disorders, consistent protective effects were found for legumes/nuts and whole grains, while fruits, vegetables, meats, dairy, and beverages demonstrated divergent patterns. Processing degree emerged as a critical determinant across all food categories in promoting sleep disorders (Supporting Information S8, Table S15).

Whole grains, legumes, and nuts consistently improved sleep through three mechanisms. First, according to Arab et al. demonstrated that magnesium and B vitamin content supports melatonin synthesis and GABA receptor function, which are essential for sleep regulation (Arab et al. 2023). Second, Xu et al. demonstrated that these foods provide anti‐inflammatory benefits by significantly reducing systemic inflammation markers (Xu et al. 2018). Third, Gangwisch et al. showed they aid glycemic control, as high glycemic index diets were associated with increased insomnia risk (Gangwisch et al. 2020). Notably, whole grain consumption protected against OSA, whereas refined carbohydrates such as white bread and porridge increased insomnia and daytime sleepiness risk (Gangwisch et al. 2020).

This detrimental pattern of processing extended across food categories. Highly palatable processed foods combining high fat, sugar, and sodium to trigger reward pathways showed harmful effects. Fatty and salty food preferences increased snoring symptoms, while savory biscuits increased daytime sleepiness. Deep processing methods including barbecuing, grilled meat and burgers were associated with increased OSA risks, potentially through advanced glycation end‐products and heterocyclic amines that promote systemic inflammation and upper airway edema (Khan et al. 2023).

Additionally, salty foods preferences emerged as risk factors for sleep disorder. According to Dayal et al., high dietary salt intake may promote fluid retention and upper airway edema, contributing to airway narrowing and increased OSA susceptibility, while also potentially inducing systemic inflammation that disrupts sleep architecture (Dayal et al. 2024). Conversely, minimally processed foods preserve natural sleep‐promoting nutrients and consistently demonstrated superior sleep benefits. These findings collectively support a fundamental principle: the less processed foods are, the more beneficial they appear for sleep disorder management and appropriate sleep duration extension. This observation extends the established inverse relationship between food processing degree and health outcomes specifically to sleep health domains.

Our results reveal dual associations between meat consumption and sleep parameters across different databases (Figures S3 and S4). Red meat, pork, and chicken consumption were associated with extended sleep duration but also with increased OSA and snoring symptoms. These patterns may be explained by distinct mechanisms: red meat, which contains high tryptophan and heme‐iron content promotes sleep duration and helps improve sleep quality (Zuraikat et al. 2021). Observational studies in older adults confirm that each additional serving of meat per week was associated with longer sleep duration (Lana et al. 2019). The protein content in meat also provides amino acids that may support sleep regulating neurotransmitter synthesis. However, the saturated fat and sodium content in red meat and chicken have been associated with systemic inflammation, fluid retention, and upper‐airway edema (Dayal et al. 2024). These physiological changes may contribute to increased OSA risk, as evidenced by correlations with higher apnea‐hypopnea indices in clinical cohorts (Kechribari et al. 2020). Processing degree further amplifies detrimental effects. Processed meat including salami, sausages, ham, and bacon exhibit shorter sleep duration and increased sleep disorder risk. Nitrite preservatives and advanced glycation end‐products promote visceral adiposity—a known OSA risk factor (Bove et al. 2018). These meat–sleep associations should be interpreted within the context of overall dietary patterns, food preparation methods, and lifestyle factors (Zuraikat et al. 2021). The dual effects suggest that meat consumption may differentially affect sleep duration versus sleep disorder risk through separate biological pathways.

For fruit and vegetables, while DASH and Mediterranean dietary patterns emphasize their overall intake can help with sleep, our findings reveal that individual food types exert distinct effects on sleep outcomes. Consistent with previous studies, strong vegetable liking benefits sleep health (Thapa et al. 2024). Vegetables broadly demonstrated protective associations against sleep disorders by reducing oxidative stress and neuroinflammation that disrupt sleep–wake regulatory circuits (İnan and Özçelik 2025). However, specific vegetable types showed divergent effects. Cabbage showed an inverse association with sleep duration and increased insomnia risk, aligning with former results (Noorwali et al. 2018). Surprisingly, our study found that pungent varieties demonstrated notable associations with sleep outcomes. Black pepper consumption reduced daytime sleepiness, likely through piperine's enhancement of dopaminergic and serotonergic neurotransmission (Haq et al. 2021). Garlic intake extended sleep duration, potentially via organosulfur compounds that reduce oxidative stress and support melatonin synthesis pathways (Askari et al. 2021). Conversely, aniseed increased daytime napping, consistent with anethole's GABAergic sedative properties (El‐Moslemany et al. 2023). As for fruits, grapefruit consumption was associated with shorter sleep duration and increased insomnia risk. In contrast, pear preference protected against short sleep. However, the underlying mechanisms by which these specific fruit components influence sleep require further investigation. These findings underscore the importance of distinguishing specific plant food components rather than relying on broad categorical classifications.

SSB demonstrated markedly different effects by beverage type. Honey reduced daytime sleepiness. Apple juice preference was associated with increased sleep duration and reduced snoring. Apple phenolic extracts—especially quercetin, catechins, and other flavonoids—have been shown in experimental studies to exert anti‐inflammatory and antioxidant effects that may attenuate neuroinflammation and oxidative stress, both implicated in sleep fragmentation (Ren et al. 2022). Conversely, fizzy drinks promoted OSA risk and daytime sleepiness as previous study (Du et al. 2021). Carbonation irritate pharyngeal mucosa, which induce low‐grade inflammation, while rapid glucose loads exacerbate fluid retention and upper‐airway edema—factors aligned with clinical observations that reducing carbonated drinks alleviates reflux‐related awakenings and snoring (Dessirier et al. 2000). Beyond our primary analyses, we identified some notable additional findings. Fat type significantly influenced sleep outcomes (Deng et al. 2022). High‐fat diet consumption, including preference for savory/caloric food, goat cheese, hard cheese, and milk chocolate, was associated with increased OSA risk and related symptoms. This aligns with evidence that high‐fat diets promote weight gain and systemic inflammation, which exacerbate upper airway collapsibility and contribute to OSA pathogenesis (Isono 2012). Dairy products, like soft cheeses, were associated with prolonged sleep duration, likely due to their high tryptophan, calcium, vitamin D, and bioactive peptides (St‐Onge et al. 2023). Fish and seafood showed contradictory evidence across datasets, likely reflecting variations in preparation methods and species‐specific nutrient profiles. Higher alcohol intake was associated with increased OSA risk in NHANES, though other databases lacked sufficient data. Alcohol may help relax pharyngeal muscles, prolonging apneas and increasing upper airway collapsibility (Peppard et al. 2007, Yang et al. 2021). Future studies should standardize alcohol intake measures for clearer analyses.

Our findings underscore two principles: food‐specific effects within categories matter, and processing degree critically determines sleep health outcomes across all food types.

Despite the comprehensive nature and methodological strengths of our multi‐database analysis, several important limitations warrant acknowledgment and suggest directions for future research. First, the cross‐sectional nature of the NHANES and GDD analyses cannot establish temporal precedence between dietary exposures and sleep outcomes. Although MR and longitudinal CLHLS data strengthen the causal arguments, unmeasured confounders may still affect the results. Future studies should prioritize prospective cohort designs with repeated dietary and sleep assessments to better capture temporal relationships and bidirectional effects (Punjabi 2008). Second, reliance on self‐reported sleep data introduces potential measurement errors and recall bias. While valuable, self‐reported measures only moderately correlate with objective assessments, such as polysomnography or actigraphy (Lauderdale et al. 2008). Future research should incorporate these objective sleep measures alongside dietary assessments for more reliable conclusions. Random controlled intervention studies comparing the effectiveness of various dietary patterns on specific sleep disorders will provide critical evidence for clinical guidelines. Further research should explore the mechanistic pathways linking dietary patterns to sleep outcomes, including inflammatory markers, oxidative stress, and metabolomics (Li et al. 2025).

## 5 Conclusion

This study addressed how dietary patterns differentially affect specific sleep‐phenotypes and identified underlying food components. This work makes three main contributions to precision sleep nutrition. First, we demonstrate that different dietary patterns offer distinct advantages for specific sleep outcomes. MED for sleep duration and sufficiency, DASH for sleep‐disordered breathing, and AHE for balanced effects across phenotypes. This challenges the “one‐size‐fits‐all” approach in dietary sleep interventions. Second, through integrated analyses combining observational and genetic data, we establish that specific food components exert differential effects: legumes, nuts, and whole grains consistently protect against sleep disorders, while the effects of other foods (fruits, vegetables, meats) vary by sleep phenotypes. Critically, food processing degree emerges as an independent determinant of sleep disorder risk beyond nutritional composition. Third, we validate these findings across diverse global populations using integrated datasets (NHANES, GDD, CLHLS, GWAS) demonstrating robustness across different ethnicities and dietary cultures. This work establishes the foundation for precision nutrition in sleep medicine. These findings enable a paradigm shift from generic dietary recommendations to phenotype specific nutritional strategies in clinical practice and public health. Future randomized trials with objective sleep monitoring and mechanistic studies integrating metabolomics and microbiome analyses are needed to establish causal pathways and further enable precision nutrition.

## Author Contributions

Ming Chen and Ke Han contributed equally to this work as co‐corresponding authors. Ming Chen and Ke Han had full access to all the data in the study and take responsibility for the integrity of the data and the accuracy of the data analysis. Meixiu Lin and Siliang Ge contributed equally to this work as co‐first authors. **Meixiu Lin:** conceptualization; data curation; formal analysis; investigation; methodology; writing – original draft; writing – review and editing. **Siliang Ge:** conceptualization; data curation; formal analysis; methodology; writing – original draft; writing – review and editing. **Kaiweisa Abuduxukuer:** conceptualization; Data curation; formal analysis; writing – original draft; writing – review and editing. **Yingfan Chen:** writing – original draft; writing – review and editing. **Shanshan Yang:** writing – original draft; writing – review and editing. **Ke Han:** conceptualization; validation; writing – review and editing; (co‐corresponding author, full data access and verification). **Ming Chen:** conceptualization; funding acquisition; validation; writing – review and editing; (co‐corresponding author, full data access and verification). All authors have read and agreed to the published version of the manuscript.

## Funding

This work was supported by the Beijing Natural Science Foundation (7254421, L222146), the National Science foundation of Liaoning Province (2025‐BS‐0946), the Liaoning Young Elite Scientists Sponsorship Program, and the National Natural Science Foundation of China (82502868, 82500816).

## Ethics Statement

The present study is an analysis of publicly available and de‐identified data and was therefore exempt from further institutional review. We utilized de‐identified data from public databases, including NHANES, GDD, CLHLS. The ethical approval was covered by the original surveys and was not necessary for the present study. Supporting Information.

## Conflicts of Interest

The authors declare no conflicts of interest.

## Supporting information


**Supporting Information: S1** Process of Sample Selection (Figure S1), NHANES Survey Weighting Procedures, Assessment of dietary components (Table S1), Assessment of Sleep Features (NHANES) (Table S2), Assessment of Covariates (NHANES) (Table S3).
**Supporting Information: S2:** Information about the Global Dietary Database (GDD) including Global Reviews on Sleep Duration (Table S4) and Global Reviews on OSA prevalence (Table S5), and Global Prevalence of OSA Cases (Figure S2).
**Supporting Information: S3:** Mendelian Randomization Data Sources and Methods (Instrumental variables for food preference with sleep‐related traits in Mendelian randomization analysis: (Table S6); MR Results: Associations of food preference with sleep‐related traits in Mendelian randomization analysis (Tables S7a) and FDR Correction Results for Mendelian Randomization Main Analyses (Table S7b.); MR Results for Processed Food and Sleep: Figure S3, and Meat and Sleep: Figure S4).
**Supporting Information: S4:** CLHLS Study Design, Sampling, Inclusion and Exclusion Criteria (Figure S5), and Variable Definitions.
**Supporting Information: S5:** Statistical Methods for Correlation Analyses in NHANES and GDD, and Causal Association Verification in CLHLS.
**Supporting Information: S6:** Scoring System Methodology (Figure 5b in the main text).
**Supporting Information: S7:** Detailed Results including Weighted logistic models evaluating the association between Dietary Patterns and OSA. (Table S8), Subgroup analysis of the relationship between Dietary Patterns and Sleep Outcome across different gender and age groups (Table S9), Data of Heatmap Analysis of Dietary Categories Associations with Sleep Health and Disorders Across Dietary Patterns (Table S10), Data of Generalized Mixed‐Effects Model Analysis of Global Dietary Category Intake with Sleep Duration (Table S11) and Data of Generalized Mixed‐Effects Model Analysis of Global Dietary Category Intake with OSA (Table S12), Association Between Dietary Factors and Sleep Outcomes in CLHLS 2008–2018 (Table S13), and Summary of the associations between dietary patterns and Sleep in NHANES (Table S14).
**Supporting Information: S8:** Summary Tables of Dietary Components and Sleep Associations Across Four Databases (Table S15), and Dietary Index Serving Size Definitions (Table S16).

**Data S1:** All Supporting Information tables and ethical approval.

## Data Availability

NHANES data are publicly available through the Centers for Disease Control (https://wwwn.cdc.gov/nchs/nhanes/Default.aspx, accessed on 23 June 2025). GDD data are publicly available through the Centers for Disease Control (https://globaldietarydatabase.org/, accessed on 23 June 2025). CLHLS data are publicly available through the PKU Center for Healthy Aging and Development (https://opendata.pku.edu.cn/dataverse/CHADS, accessed on June 23, 2025). GWAS data are available through the GWAS catalogue database (https://www.ebi.ac.uk/gwas/, Accessed on June 23, 2025) and FinnGen database (https://www.finngen.fi/en, accessed on June 23, 2025). The datasets used and/or analyzed during the current study are available from the corresponding author upon reasonable request.
