# Supplementary material for: Dietary Interventions for Sleep Health: Multi‐Population and Mendelian Randomization Evidence on Sleep Outcomes and Disorders
Source: Food Sci Nutr. 2026 Feb 10;14(2):e71475. doi: 10.1002/fsn3.71475 (PMC12896374; doi:10.1002/fsn3.71475)
Supplement: Supplementary file 1 — Supporting Information: S1 Process of Sample Selection (Figure S1), NHANES Survey Weighting Procedures, Assessment of dietary components (Table S1), Assessment of Sleep Features (NHANES) (Table S2), Assessment of Covariates (NHANES) (Table S3). Supporting Information: S2: Information about the Global Dietary Database (GDD) including Global Reviews on Sleep Duration (Table S4) and Global Reviews on OSA prevalence (Table S5), and Global Prevalence of OSA Cases (Figure S2). Supporting Information: S3: Mendelian Randomization Data Sources and Methods (Instrumental variables for food preference with sleep‐related traits in Mendelian randomization analysis: (Table S6); MR Results: Associations of food preference with sleep‐related traits in Mendelian randomization analysis (Tables S7a) and FDR Correction Results for Mendelian Randomization Main Analyses (Table S7b.); MR Results for Processed Food and Sleep: Figure S3, and Meat and Sleep: Figure S4). Supporting Information: S4: CLHLS Study Design, Sampling, Inclusion and Exclusion Criteria (Figure S5), and Variable Definitions. Supporting Information: S5: Statistical Methods for Correlation Analyses in NHANES and GDD, and Causal Association Verification in CLHLS. Supporting Information: S6: Scoring System Methodology (Figure 5b in the main text). Supporting Information: S7: Detailed Results including Weighted logistic models evaluating the association between Dietary Patterns and OSA. (Table S8), Subgroup analysis of the relationship between Dietary Patterns and Sleep Outcome across different gender and age groups (Table S9), Data of Heatmap Analysis of Dietary Categories Associations with Sleep Health and Disorders Across Dietary Patterns (Table S10), Data of Generalized Mixed‐Effects Model Analysis of Global Dietary Category Intake with Sleep Duration (Table S11) and Data of Generalized Mixed‐Effects Model Analysis of Global Dietary Category Intake with OSA (Table S12), Association Between Dietary Factors and Sleep Outco [file FSN3-14-e71475-s002.docx]

**Supplemental Information**

****Table of Contents****

[Supplementary 1--Exposure Definition in NHANES 2](#_Toc27130)

[1.1 Flowchart of the sample from NHANES selection process 3](#_Toc30819)

[1.2 Assessment of dietary metrics (NHANES) 3](#_Toc4441)

[1.3 Definitions of sleep features and covariates 4](#_Toc10836)

[Supplementary 2--Detailed Information about the Global Dietary Database (GDD) 4](#_Toc25215)

[2.1 Detailed Description of GDD 4](#_Toc25755)

[2.2 Summary of Global Reviews on Sleep Duration and OSA Prevalence 5](#_Toc677)

[2.3 Global Reviews on Sleep Duration (Supplementary TableS4). 5](#_Toc3803)

[2.4 Global Reviews on OSA prevalence 5](#_Toc31975)

[Supplementary 3--Mendelian randomization 5](#_Toc27048)

[3.1 Exoposure data 5](#_Toc29164)

[3.2 Outcome data 6](#_Toc21499)

[3.3 Sample overlapping 6](#_Toc18925)

[3.4 SNP selection(Supplementary3-TableS6) 6](#_Toc11693)

[3.5 Mendelian randomization methods(Supplementary3-TableS7a,S7b) 7](#_Toc9175)

[Supplementary 4--Cohort Study and Population in CLHLS 7](#_Toc18056)

[4.1 Study Design and Sampling 7](#_Toc21148)

[4.2 Inclusion and Exclusion Criteria 7](#_Toc1751)

[4.3 Variables Definitions 8](#_Toc497)

[Supplementary 5--Statistical Methods 9](#_Toc9646)

[5.1 Correlation analysis(NHANES) 9](#_Toc9735)

[5.2 Correlation analysis(GDD) 10](#_Toc8766)

[5.2 Causal Association Verification (CLHLS) 10](#_Toc13825)

[Supplementary 6--Scoring System（Figure5b） 10](#_Toc8430)

[Supplementary 7--Details of Results(Supplementary TableS8-14) 11](#_Toc28475)

Supplementary 8--Summary(Supplementary TableS15,16)

[Reference](#_Toc4621)

This includes:

Supplementary 1: Process of Sample Selection (Figure S1), NHANES Survey Weighting Procedures, Assessment of dietary components (Table S1), Assessment of Sleep Features(NHANES) (Table S2), Assessment of Covariates(NHANES) (Table S3).

Supplementary 2: Information about the Global Dietary Database (GDD) including Global Reviews on Sleep Duration (Table S4) and Global Reviews on OSA prevalence (Table S5), and Global Prevalance of OSA Cases (Figure S2).

Supplementary 3: Mendelian Randomization Data Sources and Methods (Instrumental variables for food preference with sleep-related traits in Mendelian randomization analysis :(Table S6); MR Results:Associations of food preference with sleep-related traits in Mendelian randomization analysis (Tables S7a) and FDR Correction Results for Mendelian Randomization Main Analyses (TableS7b.); MR Results for Processed Food and Sleep: Figure S3, and Meat and Sleep: Figure S4).

Supplementary 4: Chinese Longitudinal Healthy Longevity Survey (CLHLS) Study Design, Sampling, Inclusion and Exclusion Criteria (Figure S5), and Variable Definitions.

Supplementary 5: Statistical Methods for Correlation Analyses in NHANES and GDD, and Causal Association Verification in CLHLS.

Supplementary 6: Scoring System Methodology (Figure 5b in the main text).

Supplementary 7: Detailed Results including Weighted logistic models evaluating the association between Dietary Patterns and OSA. (Table S8), Subgroup analysis of the relationship between Dietary Patterns and Sleep Outcome across different gender and age groups (Table S9), Data of Heatmap Analysis of Dietary Categories Associations with Sleep Health and Disorders Across Dietary Patterns (Table S10), Data of Generalized Mixed-Effects Model Analysis of Global Dietary Category Intake with Sleep Duration (Table S11) and Data of Generalized Mixed-Effects Model Analysis of Global Dietary Category Intake with OSA(Table S12), Association Between Dietary Factors and Sleep Outcomes in CLHLS 2008-2018 (Table S13), and Summary of the associations between dietary patterns and Sleep in NHANES (Table S14).

Supplementary 8: Summary Tables of Dietary Components and Sleep Associations Across Four Databases (Table S15), and Dietary Index Serving Size Definitions (Table S16).

**All Supplementary tables and Ethical Approval are Uploaded in the Supplementary file (xls)).**

# Supplementary 1--Exposure Definition in NHANES

## 1.1 Flowchart of the sample from NHANES selection process


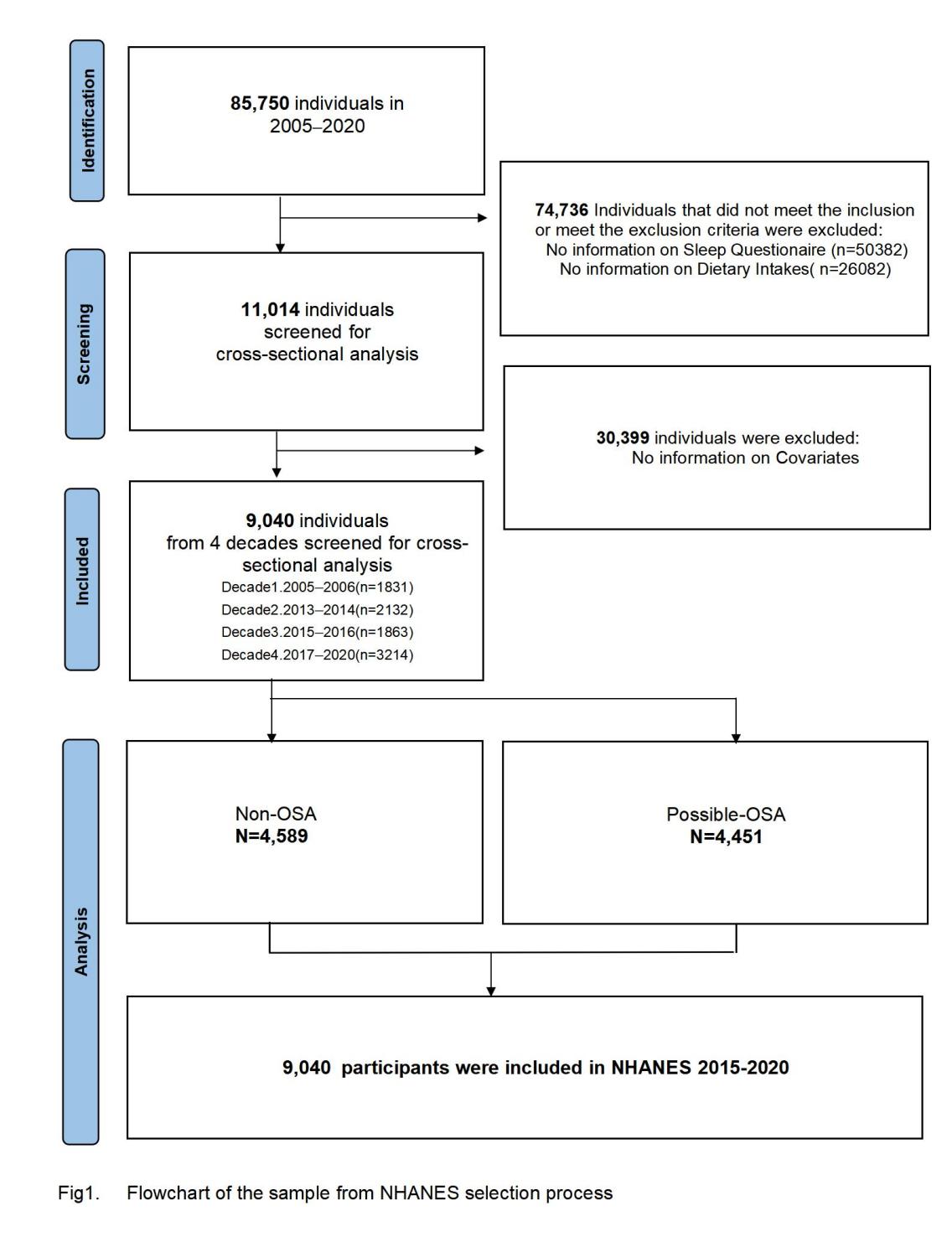


**Supplementary FigureS1**.

Flowchart of the sample From NHANES selection process

**Supplementary 1.2 NHANES Survey Weighting and Complex Design Procedures**

Following CDC/NCHS analytic guidelines, examination weights (WTMEC2YR/WTMECPRP) were selected as the appropriate sampling weights for this study. This decision is based on several methodological considerations aligned with NHANES analytic recommendations: According to CDC guidance, the sampling weight should correspond to the component where the outcome of interest was measured. Since all participants in the analytic sample completed the MEC examination to provide sleep data, WTMEC2YR/WTMECPRP (MEC examination weight) appropriately represents this study population.

Four NHANES cycles were combined. To retain national representativeness when pooling cycles of different durations, each participant's examination weight was rescaled following CDC/NCHS recommendations:

Standard cycles (2005–2006, 2007–2008, 2015–2016): Each standard 2-year cycle represents one full cycle. When pooling multiple cycles, the weight for participants from these cycles was calculated as:

We pooled 2005–2006, 2007–2008, 2015–2016, and 2017–March 2020 pre-pandemic data. Due to COVID-19, the 2017-2020 cycle was terminated after 39 months; CDC created a special pre-pandemic weight (WTMECPRP) accounting for this extended duration, equivalent to 1.625 two-year cycles (CDC/NCHS, 2021).

**Weight calculation:**

Total equivalent 2-year cycles = 3 + 1.625 = 4.625

For 2005-2006, 2007-2008, 2015-2016：1/4.625 × WTMEC2YR

For 2017-2020：1.625/4.625 × WTMECPRP

This rescaling ensures national representativeness over the combined period (CDC/NCHS, 2021). All analyses used survey-weighted procedures (survey package in R) accounting for complex sampling design (weights, stratification, clustering).

## **Supplementary 1.3** Assessment of dietary metrics (NHANES)

Dietary information was collected via two non-consecutive 24-hour dietary recalls and analyzed according to the U.S. Department of Agriculture's Food and Nutrient Database for Dietary Studies guidelines [1, 2]. Three dietary metrics (DASHI, MEDI, and AHEI) were calculated using standardized algorithms from the dietary intake data. The algorithms and R code are openly available in the dietaryindex package ([https://github.com/jamesjiadazhan/dietaryindex](https://github.com/jamesjiadazhan/dietaryindex)), a peer-reviewed and validated toolkit designed for reproducible index-based dietary pattern analysis in epidemiologic and clinical studies. This validated and flexible tool facilitates standardized calculations of dietary indices in epidemiological and clinical research[2]. Following the package workflow, computation proceeded in two steps: first, serving sizes or nutrient amounts were derived for each relevant component; second, participant-level index scores were computed from these standardized inputs[3]. Consistency with published definitions was maintained by scoring in the original units specified for each index: MEDI uses servings per day based on food weight; DASHI uses energy-adjusted nutrient densities (% energy or per 2,000 kcal); AHEI uses a mixed approach combining energy-adjusted components and absolute intake units. Complete specifications are detailed in `dietaryindex_SERVING_SIZE_DEFINITION.xlsx` (https://github.com/jamesjiadazhan/dietaryindex).

**Three representative diet quality scores were used to assess diet quality in different subjects:**
(1) **MEDI**, assessing adherence to the PREDIMED trial–derived Mediterranean diet, which emphasizes high intakes of fruits, vegetables, nuts, whole grains and olive oil as the principal fat source; moderate intakes of fish and dairy; low intakes of red and processed meats; and light to moderate wine consumption. Scored 0-12; higher scores indicate better adherence.[4]
(2) **DASHI**, the nutrient‐based DASH Index as defined by Mellen et al., comprising nine target nutrients—protein, fiber, magnesium, calcium and potassium (encouraged), and total fat, saturated fat, sodium and cholesterol (discouraged). For each nutrient, meeting the DASH trial goal yields 1 point; meeting an intermediate goal yields 0.5 points; otherwise 0 points. The sum ranges from 0 to 9, with higher scores reflecting greater adherence to the DASH dietary pattern[5].
(3) **AHEI-2010**, the Alternative Healthy Eating Index (2010 version) developed by Chiuve et al., consisting of 11 components (vegetables; fruits; whole grains; sugar-sweetened beverages and fruit juice; nuts and legumes; red/processed meat; trans fats; long‐chain (n-3) fats; polyunsaturated fats; sodium; alcohol), each scored on a 0–10 scale (0 = poorest, 10 = optimal). Component scores are summed to yield a total score from 0 to 110, with higher values indicating healthier diet quality. Trans fats were not calculated in this analysis due to data

unavailability in NHANES nutrition datasets.[6]

Assessment of dietary components are provided in **Supplementary TableS1 and relevant dietary index serving size definitions are provided in Supplementary8, Table S16**.(MEDI Definition[7]；DASHI Definition[8]；AHEI-2010 Definition [9])

## **Supplementary** 1.4 Definitions of sleep features and covariates

Sleep disorder outcomes included daytime sleepiness, obstructive sleep apnea (OSA), snoring frequency, and stop-breathing episodes. Daytime sleepiness was assessed using question SLQ120 ("How often do you feel overly sleepy during the day?"), with responses of "often" (16-30 times per month) or "almost always" (>30 times per month) classified as excessive daytime sleepiness. OSA was defined based on self-reported physician diagnosis (question SLQ030: "Have you ever been told by a doctor or other health professional that you have a sleep disorder?"). Snoring frequency was assessed using question SLQ040 ("In the past 12 months, how often did you snore while sleeping?"). Stop-breathing frequency was assessed using question SLQ060 ("In the past 12 months, how often did you stop breathing while asleep?").

Each cited study lists the NHANES sleep variables and covariates variables used and their primary focus as above. All question wordings and coding are taken from NHANES codebooks.The table reflects all available cycles and wording variations for each outcome[10, 11]Assessment of sleep features are provided in **Supplementary TableS2;** Assessment of Covariates are provided in **Supplementary TableS3**.

# Supplementary 2--Detailed Information about the Global Dietary Database (GDD)

## 2.1 Detailed Description of GDD

Dietary exposures to ten core food groups were quantified at the national level using the Global Dietary Database (GDD), which compiles harmonized, population-representative intake estimates for vegetables, fruits, nuts and seeds, whole grains, red meats, processed meats, sugar-sweetened beverages, legumes, dairy, and alcohol across 195 countries from 1990 through 2020. Country-specific mean daily intakes (g/day) were extracted and age- and sex-standardized to the World Health Organization reference population. To minimize temporal bias, dietary data were aligned with outcome data: OSA prevalence was matched to GDD estimates for **2015**, while average sleep duration was drawn from GDD estimates for **2018**. To contextualize these dietary risks within the global burden of obstructive sleep apnea (OSA), we incorporated two recent systematic reviews: Coutrot et al. (2022)[12], which delineates average sleep duration patterns across the adult life course, and Benjafield et al. (2019)[13], which provides country-level estimates of OSA prevalence and associated disability. All data processing and analyses were performed in R (v4.2.0), with dietary exposures modeled as continuous variables and OSA metrics used for cross-national comparisons of burden.

## 2.2 Summary of Global Reviews on Sleep Duration and OSA Prevalence

| Review & Year | Key Findings |
| --- | --- |
| **Coutrot et al. (2022)** | Segmented adult life into three sleep‐duration phases: early adulthood (7.5 ± 0.3 h), midlife (7.0 ± 0.2 h), late adulthood (6.5 ± 0.4 h); minimal cross‐country variance within each phase.(Supplementary2.3) |
| **Benjafield et al. (2019)** | Estimated global OSA prevalence of 1 billion adults (age 30–69) with an apnea–hypopnea index ≥5; country‐level prevalence ranged from 4% to 27%, highest in North America and Europe..(Supplementary2.4) |

## 2.3 Global Reviews on Sleep Duration (Supplementary TableS4).

## 2.4 Global Reviews on OSA prevalence

Supplementary TableS5. Global Reviews on OSA prevalence.


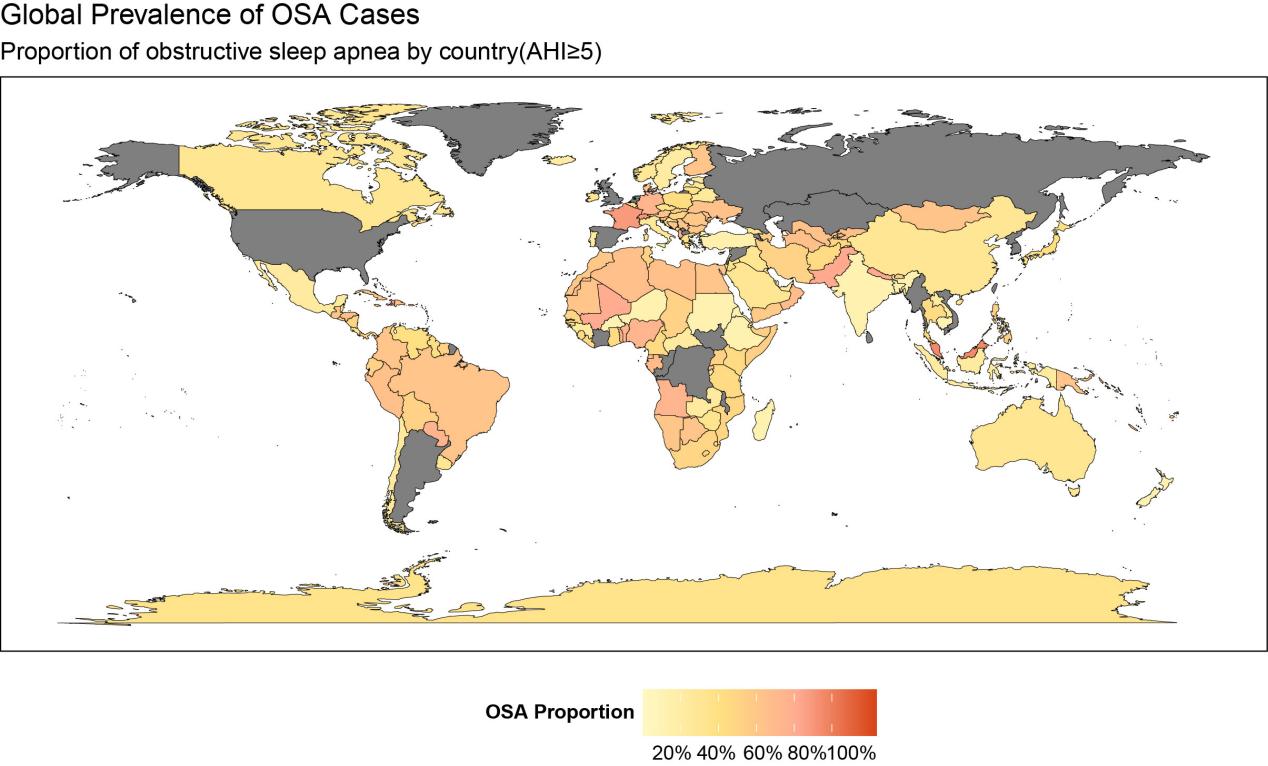


**Supplementary FigureS2.**

Global Prevalance of OSA Cases (based on Table S5)

# Supplementary 3--Mendelian randomization

## 3.1 Exoposure data

The dietary preference dataset was derived from an online questionnaire completed by over 500,000 UK Biobank participants of European ancestry[14]. This questionnaire is an expanded version of a previous one, consisting of 152 items, 139 of which are related to the food and beverages used in this study. Preferences for coffee and tea were measured both with and without sugar, generating two additional variables: the maximum preference score (coffee max and tea max) and the difference between sweetened and unsweetened scores, indicating polarization in preference.

## 3.2 Outcome data

Summary statistics for OSA were obtained from the FinnGen biobank, including 43,901 cases and 366,484 controls, with diagnoses based on ICD codes and confirmed by clinical examinations and sleep studies.

Summary statistics for self-reported sleep duration, self-reported long sleep, self-reported short duration, insomnia, snoring, daytime sleepiness and napping were all obtained from the UK Biobank.

Overall sleep duration was evaluated through self-reporting by asking participants how many hours of sleep they typically get in a 24-h period (including naps), with only integer values accepted as an answer (N = 446,118)37. Additionally, binary variables were created to distinguish short sleep duration (≤6 h vs. 7-8 h) and long sleep duration (≥9 h vs. 7–8 h). Among the individuals included in the study, 106,192 reported short sleep duration (≤6 h), 34,184 reported long sleep duration (≥9 h), and 305,742 reported a sleep duration of 7–8 h. Participants who reported using any sleep medication and those with extreme self-reported sleep durations (<3 h or >18 h) were excluded from the study[15].

Insomnia was assessed by self-reported responses to a question that inquired about difficulties falling asleep at night or waking up in the middle of the night. Participants who responded “usually” were classified as having frequent insomnia (N = 129,270), whereas those who responded “never/rarely” were considered as controls (N = 237,627)[16].

Daytime sleepiness (N = 452,071) and napping (N = 452,633) were ascertained by asking the questions “How likely are you to dose off or fall asleep during the daytime when you don’t mean to? (e.g., when working, reading, or driving)” and “Do you have a nap during the day?”, respectively[17, 18].

Data on self-reported snoring（N = 408, 317）were from a GWAS study conducted by Campos AI et al. in 2020. Snoring was assessed by asking the questions: “Does your partner or a close relative or friend complain about your snoring?” This question could be answered with “Yes”, “No”, “Don’t know”, or “Prefer not to answer”[19].

## 3.3 Sample overlapping

Because the largest, highest‐quality GWAS data for both dietary exposures and several sleep phenotypes were only available through the UK Biobank, some degree of sample overlap between exposure and outcome datasets was unavoidable. We acknowledge that overlapping samples can induce weak bias in causal effect estimates—particularly in Mendelian randomization (MR) settings, where such overlap may lead to inflated precision or biased estimates if instrument strength is modest. However, the superior data quality, rigorous phenotype definitions, and substantially larger sample sizes in UK Biobank justify its use. Accordingly, our MR framework incorporated both one‐sample and two‐sample MR approaches, with only a subset of analyses strictly adhering to two‐sample design. By leveraging these extensive UK Biobank resources, we maximized statistical power and precision while transparently noting the partial overlap as a limitation, and taking methodological precautions to mitigate its potential influence.

## 3.4 SNP selection(Supplementary3-TableS6)

The conditions for the significant association of instrumental genetic variants included: p<5×10^−8^, r2 < 0.001, a genetic distance of 10,000 kb, and all F-test values>10[20]. All phenotypes associated with the instrumental variables were examined, and SNPs related to the outcome factors (p<5×10^−8^) were excluded to ensure validity across multiple analyses in the forwards univariate MR analysis, the exposure factors consisted of 183 dietary preference data points. Specifically, SNPs that reached the genome-wide significance (P < 5 × 10^−8^) for the outcome were first removed, followed by the use of Steiger filtering to test the directionality of the association of the remaining instrumental variables with the outcome

## 3.5 Mendelian randomization methods(Supplementary3-TableS7a, S7b)

In this study, we applied inverse variance-weighted method (IVW) as our main MR model [21]. IVW assumes that all instruments are valid and thus vulnerable to horizontal pleiotropy. In cases where heterogeneity was detected, the random-effects IVW model was applied, while for all other cases, the fixed-effects IVW model was used. The weighted median, weighted mode，simple mode and MR-Egger regression methods were performed as sensitivity analyses, which hold different assumptions at the costs of reducing statistical power. If the five methods go in the same direction, the estimates are more reliable, although the simple mode, weighted median, weighted mode and MR-Egger regression are less accurate than IVW methods. Cochran's Q value was utilized to evaluate the heterogeneity among estimates from SNPs, whereas horizontal pleiotropy was assessed using the MR-Egger intercept.

## 3.6 Mendelian randomization Results

The Mendelian randomization analysis allowed us to examine meat subtypes and processing categories in greater detail and provide additional interpretation and stratified analysis for different types and processing levels of meat.


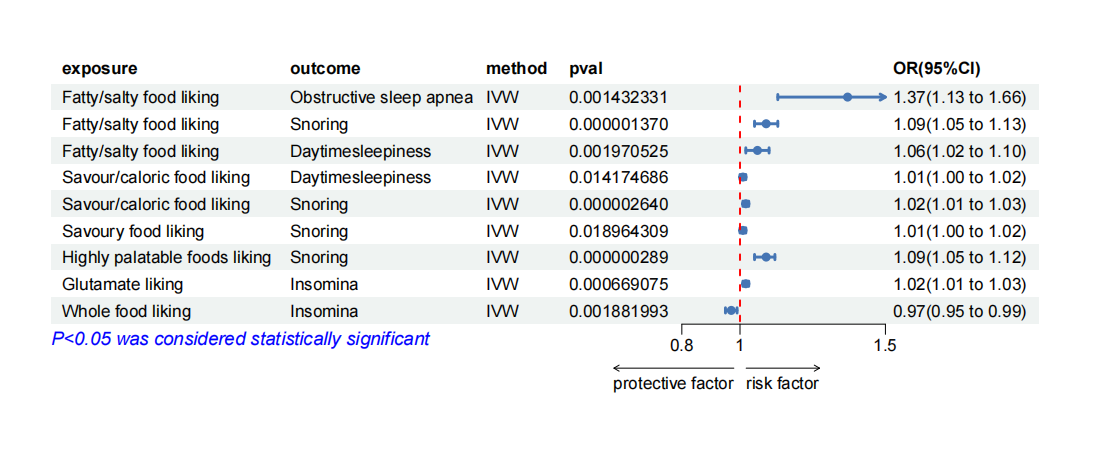


**Supplementary FigureS3.Processed Food and Sleep**


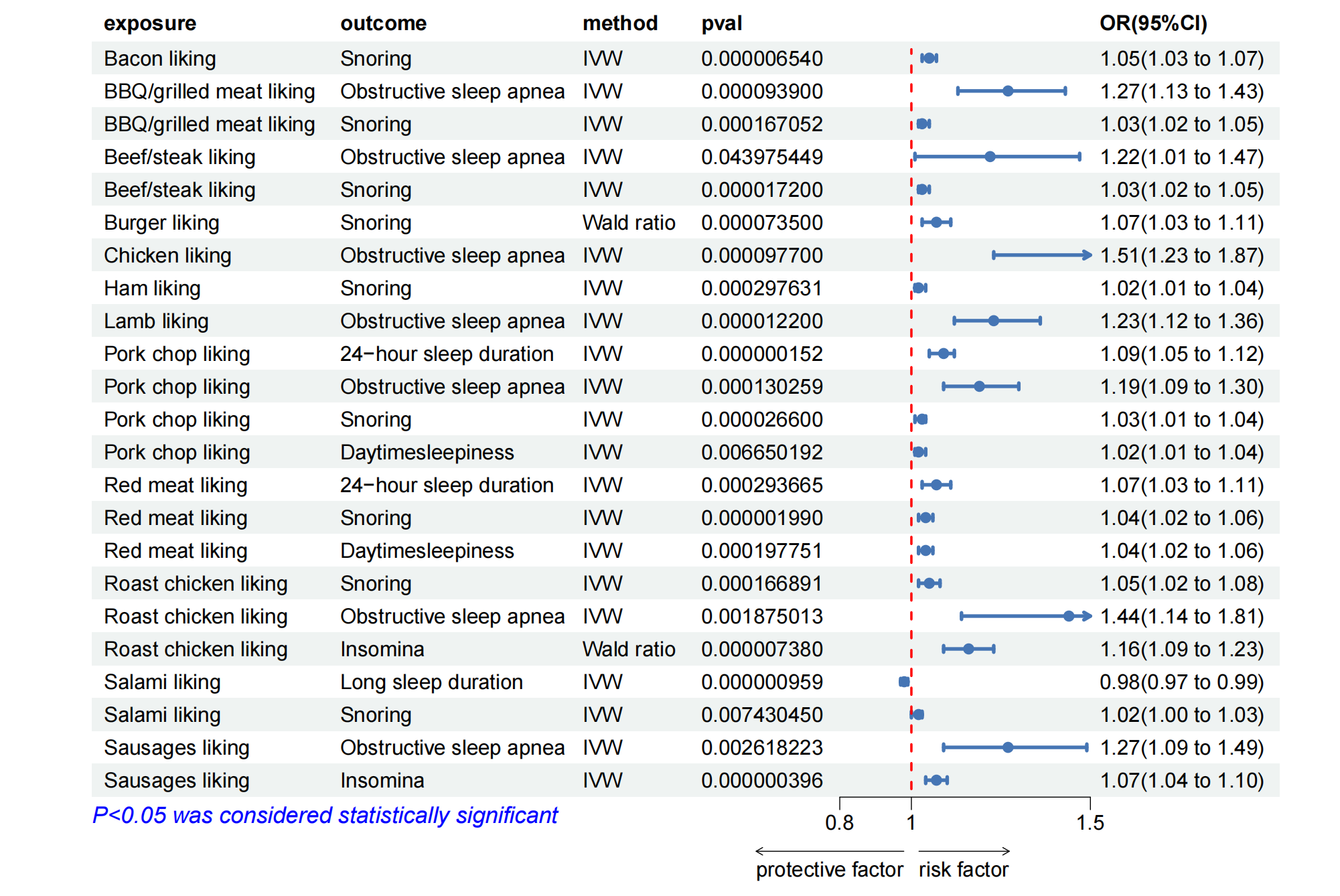


**Supplementary FigureS4. Meat and Sleep**

# Supplementary 4--Cohort Study and Population in CLHLS

**4.1 Study Design and Sampling**

The CLHLS is a nationally representative prospective cohort study established to investigate determinants of healthy aging and longevity among older adults in China. The CLHLS employs a multistage, stratified, and targeted random sampling strategy across 23 provinces, covering ~85% of China’s population. In each province, counties/cities were first selected, then townships/neighborhoods, and finally individuals aged ≥ 65 were enumerated from household registers. New participants were recruited at each wave to maintain representativeness [22].

## 4.2 Inclusion and Exclusion Criteria

**Inclusion**: Participants aged ≥ 65 at the 2008 baseline who completed both dietary and sleep modules in 2018.

**Exclusion**: Missing any of the seven dietary-frequency items at baseline or first follow-up;Missing sleep-duration or sleep-quality data at either the first or second follow-up; Missing key covariates; Loss to follow-up without outcome ascertainment .


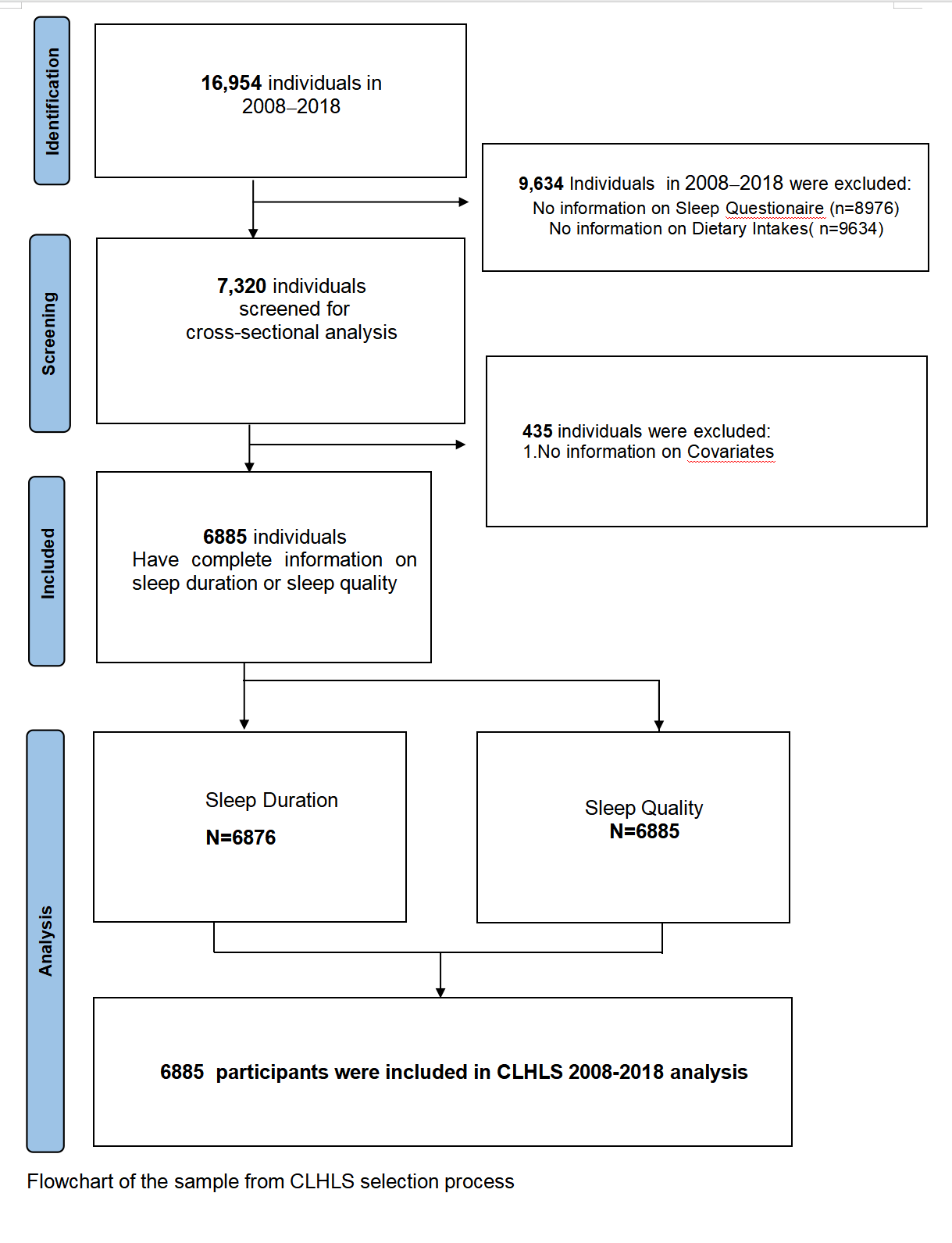
**Supplementary FigureS5.**

Flowchart of the sample from CLHLS selection Process

## 4.3 Variables Definitions

**Dietary Assessment**
 At each interview, trained interviewers asked:“Over the past year, how often did you usually eat [food group]?”

Five responses(“Rarely or never”, “Once per month”, “Once per week”, “2-3 times per week”,”Daily”)

**Sleep Measures**

**Duration:**“On average, how many hours do you sleep at night?”Participants reported integer hours; those reporting half-hours were rounded to the nearest hour.

**Quality:**“How would you rate the overall quality of your sleep at night?”--Five responses (“very good”, “good”,“so so”,“bad”,“very bad”)

**Covariate Measurement**

The covariates were selected from standardized and structured questionnaire at baseline, which included demographics, socioeconomic status, and lifestyle behaviors. Demographics included age, sex, and marital status. Socioeconomic status characteristics were measured by education level, residence area, living pattern, occupation, and household income. Measured at baseline via standardized CLHLS questionnaires, covariates included:

**Demographics**: Age (years),marital status (married vs. other)

**Socioeconomic status**: Education (none, primary, ≥ middle school), household income quartiles( Monthly per-capita household income, adjusted to 2018 RMB, then categorized into quartiles.), urban/rural residence

**Health behaviors**: Body mass index (**BMI**: Calculated as measured weight/height².During the investigation, height (cm) and weight (kg) were measured by measuring tape and weighing scale without shoes and heavy clothes.), regular physical activity (yes/no)

**Functional status**: Activities of daily living (ADL) score, assessed via Katz Index across six domains (bathing, dressing, toileting, transferring, continence, feeding), scored 0–6 [GHDx](https://ghdx.healthdata.org/record/china-longitudinal-healthy-longevity-survey-1998-2014-icpsr?utm_source=chatgpt.com).

**Cognitive function**: Mini-Mental State Examination (MMSE) score, Standard 30-point Chinese version, with < 24 indicating cognitive impairment.

# Supplementary 5--Statistical Methods

## 5.1 Correlation analysis(NHANES)

All statistical analyses were performed using R (version 4.4.0) and STATA (version 17.0). For the NHANES cross-sectional study, CDC guidelines were followed by combining multiple survey cycles and applying appropriate sampling weights . Baseline differences were assessed using t-tests for continuous variables and Chi-square tests for categorical variables.In the analysis exploring the associations between three dietary patterns (DASH, MED, and AHEI) and five sleep-related outcomes (sleep sufficiency, daytime sleepiness, OSA, stop breathing, and snoring), three logistic regression models were applied to account for increasing levels of confounding variables. Model I represents a crude model with no adjustments, while Model II controls for sociodemographic characteristics (age, gender, race, marital status, education, and income), and Model III incorporates additional adjustments for lifestyle factors such as BMI, smoking, cardiovascular diseases, stroke, cancer, liver disease, diabetes, and hypertension. Effect values are expressed as ORs with corresponding 95% CIs.Restricted cubic splines were used to examine dose-response and nonlinear relationships. Subgroup analyses assessed interactions by gender and age. P values < 0.05 were considered statistically significant. To address multiple testing across stratified analyses, we applied the Benjamini-Hochberg false discovery rate (FDR) correction with a threshold of q<0.05. P-values for interaction terms are reported for each dietary pattern-sleep outcome combination.

## 5.2 Correlation analysis(GDD)

Global dietary exposures from the Global Dietary Database (GDD) were analyzed using mixed-effects generalized linear models (MEGLM) to account for clustering of participants within continents[23]. A random intercept for continent was included in all models to adjust for unobserved regional variability.Ten dietary exposures—fruit, vegetables, red meat, seafood, sugar, dairy products, legumes, nuts, oils, and pickled vegetables—were treated as continuous predictors. Each was modeled separately. Dietary intake data were derived from the GDD and reflect median daily consumption levels at the country level.Two primary outcomes were examined: (1) the severity of obstructive sleep apnea (OSA), measured by the apnea-hypopnea index (AHI; events per hour) and dichotomized at ≥5 events/hour and (2) nightly sleep duration (hours per night), treated as a continuous variable.For AHI outcomes (count data), MEGLMs were fitted with a negative binomial distribution and a log link function. Results are presented as incidence rate ratios (IRRs) with 95% confidence intervals (CIs). For sleep duration, we used a Gaussian distribution with a log link; exponentiated coefficients represent the multiplicative change in mean sleep hours associated with each dietary exposure.Model fit and parsimony were assessed using Akaike’s Information Criterion (AIC) and Bayesian Information Criterion (BIC). All statistical analyses were conducted in Stata 17.0. A two-sided p-value < 0.05 was considered statistically significant.

## 5.2 Causal Association Verification (CLHLS)

We analyzed CLHLS data from 2008–2018 in a long-panel format (each row = person-wave), and fit individual fixed‐effects regression models for sleep outcomes. Fixed-effects (within‐person) models were used to exploit longitudinal variation in diet and sleep within individuals, thereby controlling for all time-invariant confounders[24]. In each model, the dependent variable was either sleep quality or sleep duration, and key predictors were time-varying dietary frequencies (e.g., fruits, vegetables, meats, seafoods, dairy, legumes, sugar, nuts), along with other dietary indicators (e.g., healthy oil use, bland diet, garlic, eggs, mushrooms/algae). We also included time-varying covariates (BMI, true age, marital status, economic status, physical activity, ADL score, MMSE score) to adjust for other within-person changes. Models were implemented using Stata 16.0 (xtreg, fe), and robust standard errors were used to account for heteroscedasticity[25]. This approach follows established CLHLS analyses using fixed-effects regression.

# Supplementary 6--Scoring System（Figure5b）

· **Domain-specific Scoring**: Calculate separate scores for "Sleep Health" and "Anti-Sleep Disorder"

- Sleep Health includes: sleep hour, sleep sufficiency
- Anti-Sleep Disorder includes: OSA, stop-breathing, snoring, daytime sleepiness

· **Statistical Principles**:

- Only consider filled red circles (statistically significant associations with p<0.05)
- Each symptom counts maximum 1 point, even if significant across multiple dietary patterns
- If contradictory effects appear within the same symptom or between different symptoms within the same domain, that domain scores 0 points

· **Score Calculation**:

- count +1: Each protective association (positive for sleep hour/sufficiency,negative for sleepiness/OSA/snoring/stop-breathing) ）
- count -1: Adverse associations
- Sleep Health maximum score is 2, Anti-Sleep Disorder maximum is 4

# Supplementary 7--Details of Results

Supplementary 7, Table S8. Weighted logistic models evaluating the association between Dietary Patterns and OSA.

Supplementary 7, Table S9. Subgroup analysis of the relationship between Dietary Patterns and Sleep Outcome across different gender and age groups

Supplementary 7, Table S10. Data of Heatmap Analysis of Dietary Categories Associations with Sleep Health and Disorders Across Dietary Patterns

Supplementary 7, Table S11.Data of Generalized Mixed-Effects Model Analysis of Global Dietary Category Intake with Sleep Duration

Supplementary 7, Table S12. Data of Generalized Mixed-Effects Model Analysis of Global Dietary Category Intake with OSA

Supplementary 7,TableS13.Association Between Dietary Factors and Sleep Outcomes in CLHLS 2008-2018

Supplementary 7, TableS14.Summary of the associations between dietary patterns and Sleep in NHANES

**Supplementary 8.-Summary**

Supplementary 8, Table S15 Summary of the associations between dietary components and Sleep in four database

Supplementary 8, Table S16 Summary of dietaryindex serving size definition

**Reference**

1. Bodner-Montville J, Ahuja JKC, Ingwersen LA, Haggerty ES, Enns CW, Perloff BP. Usda food and nutrient database for dietary studies: released on the web. J Food Compost Anal. 2006 2006/1/1;19:S100-07. Available from: https://www.sciencedirect.com/science/article/pii/S0889157506000147 doi: https://doi.org/10.1016/j.jfca.2006.02.002

2. Zhan JJ, Hodge RA, Dunlop AL, Lee MM, Bui L, Liang D, et al. Dietaryindex: a user-friendly and versatile r package for standardizing dietary pattern analysis in epidemiological and clinical studies. Am J Clin Nutr. 2024 2024 Nov;120(5):1165-74. Available from: http://www.ncbi.nlm.nih.gov/entrez/query.fcgi?cmd=Retrieve&db=pubmed&dopt=Abstract&list_uids=39182618&query_hl=1 doi: 10.1016/j.ajcnut.2024.08.021

3. Wang X, Yan X, Zhang J, Pan S, Li R, Cheng L, et al. Associations of healthy eating patterns with biological aging: national health and nutrition examination survey (nhanes) 1999-2018. Nutr J. 2024 2024 Sep 28;23(1):112. Available from: http://www.ncbi.nlm.nih.gov/entrez/query.fcgi?cmd=Retrieve&db=pubmed&dopt=Abstract&list_uids=39342289&query_hl=1 doi: 10.1186/s12937-024-01017-0

4. Estruch R, Ros E, Salas-Salvadó J, Covas MI, Corella D, Arós F, et al. Primary prevention of cardiovascular disease with a mediterranean diet. N Engl J Med. 2013 2013 Apr 4;368(14):1279-90. Available from: http://www.ncbi.nlm.nih.gov/entrez/query.fcgi?cmd=Retrieve&db=pubmed&dopt=Abstract&list_uids=23432189&query_hl=1 doi: 10.1056/NEJMoa1200303

5. Kling NR, Rosentrater KA, Lee D, Brellenthin AG, Lanningham-Foster L. Higher adherence to the dietary approaches to stop hypertension (dash diet) is associated with lower greenhouse gases and land use from protein foods. Front Sustain Food Syst. 2023 2023/1/1;Volume 7 - 2023. Available from: https://www.frontiersin.org/journals/sustainable-food-systems/articles/10.3389/fsufs.2023.1145272

6. Shivappa N, Hebert JR, Kivimaki M, Akbaraly T. Alternative healthy eating index 2010, dietary inflammatory index and risk of mortality: results from the whitehall ii cohort study and meta-analysis of previous dietary inflammatory index and mortality studies. Br J Nutr. 2017 2017/1/1;118(3):210-21. Available from: https://www.cambridge.org/core/product/2E9EFE57A1D2708D7F2C2DEA69FF4E16 doi: DOI: 10.1017/S0007114517001908

7. Trichopoulou A, Costacou T, Bamia C, Trichopoulos D. Trichopoulou a, costacou t, bamia c, trichopoulos dadherence to a mediterranean diet and survival in a greek population. N engl j med 348: 2599-2608. The New England Journal of Medicine. 2003 2003/7/1;348:2599-608. doi: 10.1056/NEJMoa025039

8. Liang H, Beydoun HA, Hossain S, Maldonado A, Zonderman AB, Fanelli-Kuczmarski MT, et al. Dietary approaches to stop hypertension (dash) score and its association with sleep quality in a national survey of middle-aged and older men and women. Nutrients. 2020 2020 May 22;12(5). Available from: http://www.ncbi.nlm.nih.gov/entrez/query.fcgi?cmd=Retrieve&db=pubmed&dopt=Abstract&list_uids=32455945&query_hl=1 doi: 10.3390/nu12051510

9. Al-Ibrahim AA, Jackson RT. Healthy eating index versus alternate healthy index in relation to diabetes status and health markers in u.s. Adults: nhanes 2007–2010. Nutr J. 2019 2019/1/1;18(1):26. Available from: https://doi.org/10.1186/s12937-019-0450-6 doi: 10.1186/s12937-019-0450-6

10. You Y, Chen Y, Wei M. Leveraging nhanes database for sleep and health-related research: methods and insights. Front Psychiatry. 2024 2024/1/1;15:1340843. Available from: http://www.ncbi.nlm.nih.gov/entrez/query.fcgi?cmd=Retrieve&db=pubmed&dopt=Abstract&list_uids=38745782&query_hl=1 doi: 10.3389/fpsyt.2024.1340843

11. Lee PH. Validation of the national health and nutritional survey (nhanes) single-item self-reported sleep duration against wrist-worn accelerometer. Sleep Breath. 2022 2022 Dec;26(4):2069-75. Available from: http://www.ncbi.nlm.nih.gov/entrez/query.fcgi?cmd=Retrieve&db=pubmed&dopt=Abstract&list_uids=34845630&query_hl=1 doi: 10.1007/s11325-021-02542-6

12. Coutrot A, Lazar AS, Richards M, Manley E, Wiener JM, Dalton RC, et al. Reported sleep duration reveals segmentation of the adult life-course into three phases. Nat Commun. 2022 2022 Dec 13;13(1):7697. Available from: http://www.ncbi.nlm.nih.gov/entrez/query.fcgi?cmd=Retrieve&db=pubmed&dopt=Abstract&list_uids=36509747&query_hl=1 doi: 10.1038/s41467-022-34624-8

13. Benjafield AV, Ayas NT, Eastwood PR, Heinzer R, Ip M, Morrell MJ, et al. Estimation of the global prevalence and burden of obstructive sleep apnoea: a literature-based analysis. Lancet Respir Med. 2019 2019 Aug;7(8):687-98. Available from: http://www.ncbi.nlm.nih.gov/entrez/query.fcgi?cmd=Retrieve&db=pubmed&dopt=Abstract&list_uids=31300334&query_hl=1 doi: 10.1016/S2213-2600(19)30198-5

14. May-Wilson S, Matoba N, Wade KH, Hottenga JJ, Concas MP, Mangino M, et al. Large-scale gwas of food liking reveals genetic determinants and genetic correlations with distinct neurophysiological traits. Nat Commun. 2022 2022 May 18;13(1):2743. Available from: http://www.ncbi.nlm.nih.gov/entrez/query.fcgi?cmd=Retrieve&db=pubmed&dopt=Abstract&list_uids=35585065&query_hl=1 doi: 10.1038/s41467-022-30187-w

15. Dashti HS, Jones SE, Wood AR, Lane JM, van Hees VT, Wang H, et al. Genome-wide association study identifies genetic loci for self-reported habitual sleep duration supported by accelerometer-derived estimates. Nat Commun. 2019 2019 Mar 7;10(1):1100. Available from: http://www.ncbi.nlm.nih.gov/entrez/query.fcgi?cmd=Retrieve&db=pubmed&dopt=Abstract&list_uids=30846698&query_hl=1 doi: 10.1038/s41467-019-08917-4

16. Lane JM, Jones SE, Dashti HS, Wood AR, Aragam KG, van Hees VT, et al. Biological and clinical insights from genetics of insomnia symptoms. Nat Genet. 2019 2019 Mar;51(3):387-93. Available from: http://www.ncbi.nlm.nih.gov/entrez/query.fcgi?cmd=Retrieve&db=pubmed&dopt=Abstract&list_uids=30804566&query_hl=1 doi: 10.1038/s41588-019-0361-7

17. Wang H, Lane JM, Jones SE, Dashti HS, Ollila HM, Wood AR, et al. Genome-wide association analysis of self-reported daytime sleepiness identifies 42 loci that suggest biological subtypes. Nat Commun. 2019 2019 Aug 13;10(1):3503. Available from: http://www.ncbi.nlm.nih.gov/entrez/query.fcgi?cmd=Retrieve&db=pubmed&dopt=Abstract&list_uids=31409809&query_hl=1 doi: 10.1038/s41467-019-11456-7

18. Dashti HS, Daghlas I, Lane JM, Huang Y, Udler MS, Wang H, et al. Genetic determinants of daytime napping and effects on cardiometabolic health. Nat Commun. 2021 2021 Feb 10;12(1):900. Available from: http://www.ncbi.nlm.nih.gov/entrez/query.fcgi?cmd=Retrieve&db=pubmed&dopt=Abstract&list_uids=33568662&query_hl=1 doi: 10.1038/s41467-020-20585-3

19. Campos AI, García-Marín LM, Byrne EM, Martin NG, Cuéllar-Partida G, Rentería ME. Insights into the aetiology of snoring from observational and genetic investigations in the uk biobank. Nat Commun. 2020 2020 Feb 14;11(1):817. Available from: http://www.ncbi.nlm.nih.gov/entrez/query.fcgi?cmd=Retrieve&db=pubmed&dopt=Abstract&list_uids=32060260&query_hl=1 doi: 10.1038/s41467-020-14625-1

20. Palmer TM, Lawlor DA, Harbord RM, Sheehan NA, Tobias JH, Timpson NJ, et al. Using multiple genetic variants as instrumental variables for modifiable risk factors. Stat Methods Med Res. 2012 2012 Jun;21(3):223-42. Available from: http://www.ncbi.nlm.nih.gov/entrez/query.fcgi?cmd=Retrieve&db=pubmed&dopt=Abstract&list_uids=21216802&query_hl=1 doi: 10.1177/0962280210394459

21. Burgess S, Scott RA, Timpson NJ, Davey SG, Thompson SG. Using published data in mendelian randomization: a blueprint for efficient identification of causal risk factors. Eur J Epidemiol. 2015 2015 Jul;30(7):543-52. Available from: http://www.ncbi.nlm.nih.gov/entrez/query.fcgi?cmd=Retrieve&db=pubmed&dopt=Abstract&list_uids=25773750&query_hl=1 doi: 10.1007/s10654-015-0011-z

22. Zeng Y. Towards deeper research and better policy for healthy aging --using the unique data of chinese longitudinal healthy longevity survey. China Economic J. 2012 2012/1/1;5(2-3):131-49. Available from: http://www.ncbi.nlm.nih.gov/entrez/query.fcgi?cmd=Retrieve&db=pubmed&dopt=Abstract&list_uids=24443653&query_hl=1 doi: 10.1080/17538963.2013.764677

23. Miller V, Reedy J, Cudhea F, Zhang J, Shi P, Erndt-Marino J, et al. Global, regional, and national consumption of animal-source foods between 1990 and 2018: findings from the global dietary database. Lancet Planet Health. 2022 2022 Mar;6(3):e243-56. Available from: http://www.ncbi.nlm.nih.gov/entrez/query.fcgi?cmd=Retrieve&db=pubmed&dopt=Abstract&list_uids=35278390&query_hl=1 doi: 10.1016/S2542-5196(21)00352-1

24. Wang Y, Yang W. Does receiving informal care lead to better health outcomes? Evidence from china longitudinal healthy longevity survey. Res Aging. 2022 2022 Aug-Sep;44(7-8):510-18. Available from: http://www.ncbi.nlm.nih.gov/entrez/query.fcgi?cmd=Retrieve&db=pubmed&dopt=Abstract&list_uids=34772286&query_hl=1 doi: 10.1177/01640275211052834

25. Wang X, Miao H, Jin Q. The structural characteristics of the lifestyle among older adults and its impact on the health in china. Front Public Health. 2023 2023/1/1;11:1286530. Available from: http://www.ncbi.nlm.nih.gov/entrez/query.fcgi?cmd=Retrieve&db=pubmed&dopt=Abstract&list_uids=38155896&query_hl=1 doi: 10.3389/fpubh.2023.1286530
